# Field Efficacy of Larvivorous Fish and Pyriproxyfen Combined with Community Engagement on Dengue Vectors in Cambodia: A Randomized Controlled Trial

**DOI:** 10.4269/ajtmh.20-1088

**Published:** 2021-09-07

**Authors:** John Christian Hustedt, Dyna Doum, Vanney Keo, Sokha Ly, BunLeng Sam, Vibol Chan, Neal Alexander, John Bradley, Marco Liverani, Didot Budi Prasetyo, Agus Rachmat, Muhammad Shafique, Sergio Lopes, Leang Rithea, Jeffrey Hii

**Affiliations:** ^1^ Epidemiology Department, Malaria Consortium, Phnom Penh, Cambodia;; ^2^ MRC Tropical Epidemiology Group, Department of Infectious Disease Epidemiology, London School of Hygiene and Tropical Medicine, London, United Kingdom;; ^3^ Department of Vector Control, Cambodian National Dengue Control Program, Phnom Penh, Cambodia;; ^4^ Department of Climate Change and Health, World Health Organization, Phnom Penh, Cambodia;; ^5^ Department of Global Health and Development, London School of Hygiene and Tropical Medicine, London, United Kingdom;; ^6^ School of Tropical Medicine and Global Health, Nagasaki University, Nagasaki, Japan;; ^7^ Department of Entomology, US Naval Medical Research Unit-2, Phnom Penh

## Abstract

Evidence on the effectiveness of low-cost, sustainable biological vector control tools for *Aedes* mosquitoes is limited. Therefore, the purpose of this trial was to estimate the impact of guppy fish in combination with the larvicide pyriproxyfen (PPF) (Sumilarv^®^ 2MR) and communication for behavioral impact (COMBI) activities to reduce entomological indices in Cambodia. In this cluster randomized, controlled superiority trial, 30 clusters comprised of one or more villages each was allocated in a 1:1:1 ratio to receive either 1) all three interventions (guppies, PPF, and COMBI), 2) two interventions (guppies and COMBI), or 3) control (standard vector control). Entomological surveys among 40 randomly selected households per cluster were carried out quarterly. The primary outcome was the population abundance of adult female *Aedes* mosquitoes trapped using adult resting collections. In the primary analysis, adult female *Aedes* abundance and mosquito infection rates was aggregated over follow-up time points to give a single rate per cluster. These data were analyzed by negative binomial regression, yielding abundance ratios (ARs). The number of *Aedes* females was reduced roughly by half compared with the control in both the guppy, PPF, and COMBI arm (AR = 0.54; 95% CI, 0.34–0.85; *P* = 0.0073); and the guppy and COMBI arm (AR = 0.49; 95% CI, 0.31–0.77; *P* = 0.0021). The effectiveness demonstrated and extremely low cost of including fish rearing in community-based health structures suggest they should be considered as a vector control tool as long as the benefits outweigh any potential environmental concerns. Sumilarv^®^ 2MR was also highly accepted and preferred over current vector control tools used in Cambodia.

## INTRODUCTION

Dengue is the most rapidly spreading mosquito-borne viral disease in the world and is caused by bites of infected *Aedes* mosquitoes, principally *Aedes aegypti*.^1^ Dengue is concentrated in the Asian region, which shoulders 70% of the global disease burden. Although a number of promising vaccine candidates are in preclinical and clinical development,^2^ innovative methods of genetic control of mosquitoes are being developed^3^^–^^6^; however, these interventions are unlikely to eliminate dengue on their own.^7^ Therefore, traditional vector control remains a key component of dengue control in the short and medium terms.

In Cambodia, 194,726 dengue cases were reported to the National Dengue Control Program (NDCP) between 1980 and 2008.^8^ However, the real number of cases and cost to society is estimated to be many times higher.^9^^,^^10^ Previous work showed household water storage jars contained more than 80% of *Ae. aegypti* larvae in Cambodia, and these jars became the main target for dengue vector control activities.^11^

Since the early 1990s, NDCP has used the larvicide temephos (Abate**^®^**, Ludwigshafen, Germany) to target large (200–400 L) household water containers as the primary means of vector control.^12^ This has continued despite tests published in 2001, 2007, and 2018 showing resistance of *Ae.* a*egypti* in several provinces across Cambodia.^12^^–^^14^ Khun and Manderson^12^ concluded that “continued reliance on temephos creates financial and technical problems, while its inappropriate distribution raises the possibility of larvicide resistance.” These problems led researchers to consider alternative control methods, including chemical and biological substances (pyriproxyfen [PPF] and *Bacillus thuringiensis israelensis*),^1^^,^^12^^,^^15^^,^^16^ jar covers,^11^ and the distribution of larvivorous copepods and fish.^17^^–^^19^ The interventions that had the most effective results included the use of larvivorous fish and PPF.^1^^,^^17^

The use of a larvivorous guppy (*Poecilia reticulata*) was evaluated in 14 Cambodian villages,^19^ and subsequently in a larger study of 28 Cambodian villages.^17^ Results from the initial study conducted from 2006 to 2007 were encouraging, because even with a low coverage of guppies (in 56% of eligible containers 1 year after project commencement) there was a 79% reduction in *Aedes* infestation compared with the control area. Despite not having guppies, the smaller or discarded containers in the intervention area had 51% less infestation than those in the control area, suggesting a community-wide protective effect.^19^ These results led the WHO and the Asian Development Bank to fund a larger scale-up in 2010 and 2011 that included communication for behavioral impact (COMBI) activities. At the end of the implementation period, an evaluation found that 88% of water jars, tanks, and drums contained guppy fish, suggesting successful establishment of breeding sites. In addition, the container index (the percentage of water holding containers infested with *Aedes* larvae or pupae) and the number of indoor resting adult females in the intervention area were near zero, whereas the control area had a container index of 30.^17^ Similarly encouraging results were found in Laos as a part of the same project, although many water containers in the implementation area were too small for guppy survival. This experience indicates that additional tools beyond larvivorous fish are required to target smaller water containers as well as hard-to-reach and cryptic breeding sites.

One potential solution to increase coverage of water containers in the communities is the use of PPF, a juvenile hormone analog that interferes with the metamorphosis of juvenile *Aedes* mosquitoes, preventing their development. It can be used in small or contaminated containers unsuitable for larvivorous fish.^20^ Studies of the efficacy of PPF in Cambodia showed inhibition of adult emergence greater than 87% for 6 months in 2003,^15^ and inhibition of adult emergence of more than 90% for 20 weeks and more than 80% for 34 weeks in 2007.^1^ A slow-release PPF matrix release formulation (Sumilarv^®^ 2MR) has been developed and shown to be effective in Myanmar.^21^ This new product only requires one distribution every 6 months (the entirety of the rainy season), so it reduces operational costs compared with temephos or *Bti*, which have a residual efficacy of 2 to 3 months.^16^^,^^22^

Yet the efficacy of these measures, like other vector management approaches in the communities, is not only dependent on their entomological efficacy, but also requires mobilization and coordination of resources to sustain behavior changes.^23^ In particular, a key challenge for vector control in the communities is how local residents can be involved in and sustain vector breeding source reduction efforts.^17^ Recent reviews indicate that a strong communication and behavior change approach, such as COMBI, has the potential to support vector management programs with very good outcomes.^24^^,^^25^ For example, two new cluster randomized trials found that educational messages embedded in a community-based vector control approach were effective at reducing *Ae. aegypti* measured through entomological indices.^26^^,^^27^

### Need for a trial.

Although there is evidence suggesting the use of guppy fish can be beneficial in dengue vector control, recent reviews show there has never been a cluster randomized trial to evaluate their effect on mosquito indices.^28^ This trial has the potential to inform the strategic application of community-based distribution of PPF and larvivorous fish in an outbreak, during inter-epidemic periods or for broad-scale application. This trial is also the first (to our knowledge) to evaluate the widescale use of the new Sumilarv 2MR product in the field. Furthermore, PPF and larvivorous fish have never been tested in combination. Our study is intended to fill these knowledge gaps.

### Hypothesis.

This trial aimed to demonstrate community effectiveness of guppies, PPF, and COMBI activities. There are three main hypotheses. First, use of guppies, Sumilarv 2MR, and COMBI activities will reduce numbers of *Aedes* mosquitoes, and their infection rates, more than guppies and COMBI alone or standard vector control activities (such as larval control and information and education material dissemination during outbreaks) as assessed through entomology surveys. Second, COMBI activities will improve the community’s knowledge, attitude, and behavior related to water use and vector-borne disease prevention (such as burning or burying discarded containers, cleaning the environment around the house, and sleeping under a bed net) as assessed through baseline/end-line surveys and focus group discussions (FGDs). And third, guppies and PPF will be acceptable among the target villages as assessed by an end-line survey and FGDs.

## MATERIALS AND METHODS

This study followed the Consolidated Standards of Reporting Trials guidelines^29^ (Supplemental Table 1).

### Study design and setting.

The study was designed as a cluster randomized, controlled trial with three arms. The study had 30 clusters in the province of Kampong Cham, where each cluster was a village or group of villages with 170 households on average (range, 49–405) or 757 individuals (range, 250–1,769). The rainy season runs from April to November, and the peak dengue season is from May to July. The clusters were selected in areas that had *Aedes* infestation in the past. To minimize potential spillover effects, clusters had to be at least 200 m from the nearest household outside the cluster, because *Ae. aegypti* in this region have an average flight range of 50 to 100 m.^30^ Every house within the cluster boundaries was invited to participate in the trial.

### Interventions.

Selected villages were randomized into one of three study arms (Table 1). Reasons for selecting the interventions for each arm are described earlier and in more detail in the study protocol.^31^ During the length of the trial, no vector control interventions were completed in the study area by the government or any other government-linked actor outside the study team. Individuals may have purchased repellent products from the market, but no items were purchased or distributed by outside funds. The total trial period for the interventions was 11 months (Supplemental Figure 1).

**Table 1:** Interventions randomized to each study arm

Intervention	Arm 1 (guppy, COMBI, and PPF)	Arm 2 (guppy and COMBI)	Arm 3 (control)
Guppy fish in key containers (> 50 L)	X	X	–
COMBI activities	X	X	–
Direct PPF application (Sumilarv^®^ 2MR) in smaller containers (10–50 L)	X	–	–

COMBI = communication for behavioral impact; PPF = pyriproxyfen.

#### Guppies.

Two guppy fish (*Poecilia reticulata*) were placed into each water container larger than 50 L in intervention villages (arms 1 and 2). This was based on larval consumption of guppies determined by Seng et al.^11^ and past experiences using guppies as a vector control in Cambodia.^17^ The guppies were sourced from the original NDCP colony, which was started from guppies found in a rural waterway near Phnom Penh roughly 15 years earlier. The guppy fish were distributed after the baseline activities through a local community network managed by provincial government authorities.^31^ Community health workers (CHWs) were provided two jars for rearing. Each month, CHWs conducted visual checks and ensured all their assigned households had guppies in all large containers and replaced them if necessary.

#### PPF matrix release (Sumilarv 2MR).

The product contains PPF in an ethylene copolymer resin disk, and the PPF is released gradually from the polymer material until it reaches an equilibrium state of the dissolved active ingredient with that in the matrix formulation.^32^ Each device is designed to provide coverage for 40 L water, and can be cut into smaller sizes for smaller containers.^31^ PPF devices were distributed to 10- to 50-L containers at the beginning of the trial and were replaced after 6 months. Additional devices were left at the health center (HC) for CHWs to distribute during their monthly monitoring visit if some were lost or needed to be replaced. The exceptional safety of PPF is reflected in the WHO’s statements that it is “unlikely to present acute hazard in normal use,” “pyriproxyfen does not pose a carcinogenic risk to humans,” and PPF “is not genotoxic.”^33^ As a result of its efficacy, the WHO Pesticide Evaluation Scheme has recommended the use of PPF for mosquito control.^34^ Animal models suggest a very favorable mammalian toxicity profile and extremely low risk for humans using this product.^35^

#### COMBI activities.

An initial rapid assessment consisting of FGDs and in-depth interviews regarding knowledge, attitudes, and behaviors of community members was completed. The results were used in a message and material development workshop held with key community and district stakeholders. During this meeting, the community helped to develop behavior change communication materials and come up with key messages. The results were used to understand the common social gathering locations for health education sessions and culturally appropriate channels of communication, and to create communication materials: flip charts to guide CHW education sessions, posters and banners for display in the villages, songs, and CHW materials such as hats, t-shirts, bags, and raincoats.

A 2-day training was given to CHWs on communication and facilitation skills, after which the CHWs took the lead role in conducting health education sessions in their community. Monthly meetings were also conducted with CHWs to assess progress, address issues and challenges, and provide them with continuous training to develop their confidence and skills. The health education sessions occurred twice per month and were participatory, as Khun and Manderson^36^ found that health education sessions, during which participants actively identify breeding sites and practice positive behaviors, can be more effective and less costly than the didactic classroom-based sessions. In addition to health education sessions, locally available media, such as loudspeakers fixed to local transport to play songs, and role-playing were used to reinforce the messages.

### Adherence.

To improve adherence to the intervention protocols, CHWs performed monthly monitoring checks on each household within the intervention arms, and entomological surveys were used to record the presence or absence of each intervention in containers.^31^ Project staff also visited CHWs and intervention households randomly to confirm the reliability of data provided.

### Primary outcome measures.

The primary outcome measure was the population abundance (i.e., number of mosquitoes per unit of time spent aspirating) of adult female *Aedes* trapped using adult resting collections.

### Secondary outcome measures.

The secondary outcomes for the trial included 1) dengue virus infection rate in adult female *Aedes* mosquitoes; 2) house index, which was the proportion of houses surveyed positive for *Aedes* larvae and/or pupae in any water container; 3) container index, which was the proportion of surveyed containers containing *Aedes* larvae and/or pupae; 4) the Breteau index, which was the number of containers positive for *Aedes* larvae and/or pupae per 100 houses surveyed; 5) the number of *Aedes* pupae per household; 6) the number of *Aedes* pupae per person; 7) guppy fish coverage, which was the proportion of eligible water containers with ≥ 1 guppy fish; 8) Sumilarv 2MR coverage, which was the proportion of eligible water containers with ≥ 1 MR resin disk; and 9) the percentage of respondents with knowledge about *Aedes* mosquitoes causing dengue.

### Sample size.

The guppy fish and PPF interventions were assessed using four entomological surveys. A sample size of 10 clusters per arm and 40 households per cluster for the survey was devised using the Hemming and Marsh method,^34^ assuming a mean of 0.1 adult female resting *Aedes* per household in the intervention arms compared with 0.25 in the control arm for each collection based on previous studies. The households were selected randomly during each collection. The intra-cluster correlation was assumed to be 0.01 based on previous studies.^35^^–^^37^ In addition, a sensitivity analysis was conducted up to the median value of intra-cluster correlations for outcome variables (0.03) as found by an analysis conducted by Campbell et al.^38^ Our analysis determined that intra-cluster correlation values between 0.01 and 0.03 would have 91% and 75% power, respectively.

The impact of COMBI activities in the communities was evaluated through knowledge, attitudes, and practice (KAP) surveys. A sample size of 10 clusters per arm and 20 households per cluster was devised, again using the Hemming and Marsh method,^34^ assuming a 22.5% change in KAP indicators from 40% to 62.5% in intervention villages and no change in the control villages over the course of 1 year.^31^

### Allocation.

Clusters were assigned randomly with a 1:1:1 allocation through a public randomization process. Village chiefs from all clusters and HC chiefs from all HCs were invited to a central point, along with local and national authorities, where allocation took place. Allocation concealment was accomplished by having each cluster representative choose one folded-up paper with a printed label referring to arms 1, 2, or 3.

### Data collection methods.

Data were collected at 0, 4, 8, and 12 months post-intervention, unless otherwise mentioned. The timing was also meant to capture data over different season (heavy rain, light rain, and dry seasons). The project used the methods discussed in the following subsections.

#### Entomology.

A baseline survey was conducted prior to the start of interventions. An end-line survey was conducted 1 year after the baseline. Two additional surveys during the dry season (4 months post-intervention) and light rain (8 months post-intervention, peak dengue season) were also conducted. The survey methodology was developed according to WHO guidelines for entomological collections^42^ and are detailed in the study protocol.^31^ The adult resting catch was completed using a battery-powered portable aspirator (Camtech, Phnom Penh, Cambodia) for 10 min/house in the bedrooms and living spaces, starting in the bedroom and aspirating up and down the wall (from floor to 1.5 m) around the home in a clockwise manner. The survey team also used a rapid assessment tool—the premise condition index)^40^—to identify whether the scores can predict household risk for *Ae. aegypti* infestation.^41^

#### Knowledge, attitudes, and practices.

The KAP surveys were conducted at the same time as the baseline and end-line entomological surveys.^31^ The secondary outcome measure included was whether participants knew dengue is transmitted by mosquitoes of the genus *Aedes*, rendered in Khmer as “kala”—meaning, feline or tiger.

#### CHW monthly monitoring.

The coverage of guppy fish and PPF Sumilarv 2MR was assessed by ocular inspection of water containers via entomological surveys and the CHW monthly reporting form as described in “Adherence.” Coverage is expressed as the percentage of containers with at least two guppy fish or one Sumilarv 2MR of the total households or containers examined.

#### Climate.

General climate data (rainfall, temperature, and humidity) were recorded at one of the intervention health centers using a rain gauge and a Hobo onset data logger. All villages have virtually the same climate.

#### Data management.

The first two entomological surveys and the first KAP survey were recorded on paper, and double data entry was performed using EpiData (EpiData Association, Denmark) by an experienced data processing company. As a result of factors including budget, timeliness, and need for data cleaning, the subsequent two entomological surveys and final KAP survey were recorded electronically on Samsung tablets (Samsung Group, South Korea) and the data were uploaded to ONA servers.

### Mosquito flavivirus infection.

Adult female *Aedes* mosquitoes were pooled together by cluster, with a maximum of 10 per pool, and an expected minimum infection rate of 3% to 7% based on other studies.^42^^,^^43^ Flavivirus detection in adult female mosquitoes followed the protocol set out by Pierre et al.^44^ using a set of universal oligonucleotide primers. Samples identified as positive for flavivirus were then put into a rapid assay for detecting and typing dengue viruses.^45^ All pools had positive and negative controls to ensure the tests were working properly.

### Statistical methods.

All statistical analyses were performed in R (v. 3.5.0; Murray Hill, NJ) and Stata^®^ (v. 14.2; Stat Corp., (College Station, TX).

#### Primary outcome.

Adult female *Aedes* abundance was summed over follow-up time points to give a single rate per cluster. This was analyzed by negative binomial regression using the number of adults as the response and the logarithm of the sampling effort (that is, person-time spent aspirating) as an offset. Hence, this analysis yielded abundance ratios (ARs).

#### Secondary outcomes.

None of the mosquito pools tested were positive for dengue virus; consequently, the minimum infection rate was 0%. The most commonly used entomological indexes (Breteau index and pupae per person) are reported here; correlated indices (container index, house index, and pupae per house) are listed in Supplemental Table 2.

### Data monitoring.

In accordance with the recommendations of Grant et al.,^46^ we did not establish a data safety monitoring board for this study because it is not a “clinical trial evaluating a therapy with a mortality or irreversible morbidity endpoint.” However, a technical steering committee was established and met at least every 6 months to address any concerns that arose.^31^ Participants were told to report any adverse events directly to project staff or CHWs and to seek medical attention immediately. CHW monthly monitoring forms included a line to report any adverse events that took place. Any report of harm or adverse events was reported directly to the technical steering committee.

### Access to data.

All co-principal investigators and partners were given access to the cleaned data sets without identifiers, which were stored on the Malaria Consortium Sharepoint site and were password protected. The final anonymized data set will be stored in the Cambodian National Center for Parasitology, Entomology, and Malaria Control central repository. The final cluster-level data set used for the analysis in “Results” is included as supplemental material. Entomological specimens are stored for 2 years at Malaria Consortium offices should other researchers be interested in accessing them.

### Blinding.

The data collectors were not blinded in this case because teams were able to see the interventions in the containers they sampled. The data collectors were government staff and therefore had access to the protocol when submitting to the ethics committee. The staff members doing the analysis were also involved in data collection and therefore were not blinded.

### Ethical approval and consent to participate.

Ethical clearance for this trial was received by the Cambodian National Ethics Committee for Health Research on October 9, 2014 (ethics reference no. 0285). In addition, ethics approval was received from the London School of Hygiene and Tropical Medicine Observational/Interventions Research Ethics Committee (ethics reference no. 8812). CHWs explained the trial and received informed consent from the head of the household before providing the interventions.^31^ Those who were illiterate or otherwise could not sign their name were given the option of providing their thumbprint. During the informed consent process, all participants were told that, at any point, they could choose to stop using any of the items and remove themselves from the study. If participants wanted to leave the study, they simply informed the village malaria workers. All village and respondent names were deleted to ensure no identifying information was included. Data from surveys were stored in a password-protected computer. All qualitative data were collected in concordance with the guidelines of the Code of Ethics of the American Anthropological Association.^47^

## RESULTS

### Baseline results.

The control arm of the baseline results had a slightly larger number of houses/people than the intervention arms (Table 2). The gender and age distribution of household heads was similar among the three arms. The mean number of containers, positive containers, Breteau index, and pupae per person at cluster level were all larger in the guppy-only arm (arm 2) than others, whereas the mean number of adult *Aedes* females per cluster was similar among arms.

**Table 2:** Baseline summary measures of containers, houses, and people per cluster

Summary measures	Control	Guppies	PPF + Guppies
Clusters, *n*	10	10	10
Houses, *n*	2,016	1,641	1,435
People, *n*	8,475	7,542	6,700
Houses surveyed, *n*	400	400	400
Male household heads, % (range)	22 (10–45)	23 (10–32)	20 (10–35)
Median age of household head, y (range)	42 (17–78)	42 (18–84)	45 (18–88)
Containers per cluster, *n* (range)	154 (121–190)	186 (160–219)	165 (110–213)
Mean number of positive containers per cluster* (range)	24.7 (18–62)	36.5 (18–62)	27.7 (11–69)
Mean Breteau index per cluster (range)	62 (20–115)	91 (45–155)	69 (28–173)
Mean pupae per person, *n* (range)	0.9 (0.2–2.7)	4.0 (0.2–17.1)	1.1 (0.5–2.3)
Mean adult *Aedes* female abundance per cluster, *n *(range)	10 (1–15)	9 (3–24)	11 (2–20)

PPF = pyriproxyfen.

* Positive is defined as having either *Aedes* pupae or larvae in the container.

### Primary outcome.

**Figure 1.**
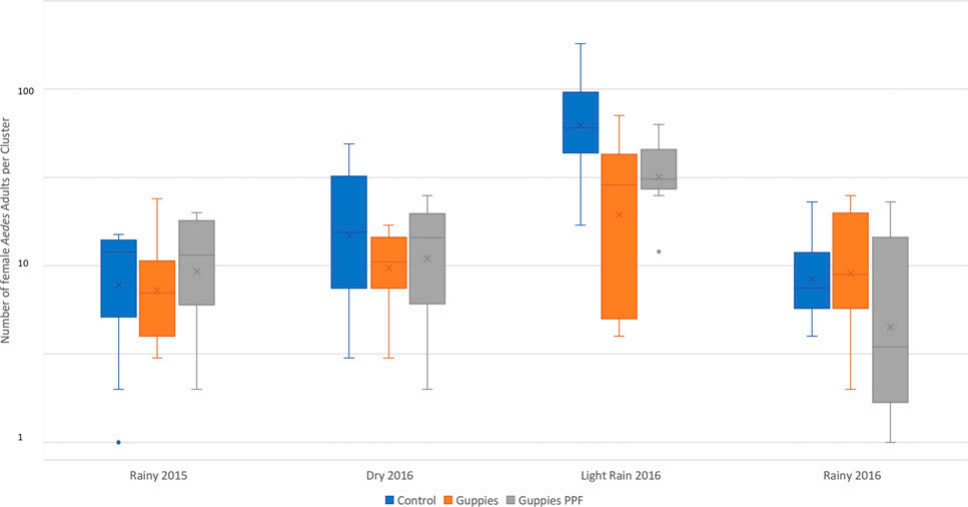
Box plots showing mean number of adult *Aedes* females per household by arm and season, October 2015 to October 2016. The population abundance (i.e., number of mosquitoes per unit of time spent aspirating) was determined using adult resting collections. This figure appears in color at www.ajtmh.org.

During the intervention period, the population abundance of adult female *Aedes* was significantly less in both intervention arm 1 (guppies, PPF, and COMBI) (AR = 0.54; 95% CI, 0.34–0.85; *P* = 0.0073) and arm 2 (guppies and COMBI) (AR = 0.49; 95% CI, 0.31–0.77; *P* = 0.0021) relative to arm 3 (control). However, the difference between the two intervention arms was not significant (AR = 1.10; 95% CI, 0.69–1.74; *P* = 0.6901) (Table 3). The mean number of adult *Aedes* females was highest during the light-rain season and lowest in the rainy season (Figure 1).

**Table 3:** Mean population abundance of adult female *Aedes* trapped using adult resting collections per cluster by arm and survey

	Control	Guppies	Guppies + PPF
Season			
Baseline (range)	10 (1–15)	9 (3–24)	11 (2–20)
Dry season (range)	20 (3–49)	11 (3–17)	14 (2–25)
Light rain (range)	75 (17–181)	29 (4–71)	35 (12–63)
Heavy rain (range)	10 (4–23)	12 (2–25)	8 (1–23)
Total (range)	35 (3–181)	17 (2–71)	19 (1-63)
Abundance ratio			
Abundance ratio (95% CI), *P* value*	1 (ref)	0.49 (0.31–0.77), *P* = 0.0021	0.54 (0.34–0.85), *P* = 0.0073
Abundance ratio (95% CI), *P* value*	†	1 (ref)	1.10 (0.69–1.74), *P* = 0.6901

PPF = pyriproxyfen. The trapping time was 10 minutes per house.

* The ratios do not include the baseline data.

† The ratio is not given here because it would be redundant.

### Secondary outcomes.

No adult female *Aedes* mosquitoes in any arm was found to be positive by polymerase chain reaction for dengue virus (*N* = 280 pools). The most commonly used entomological indexes (Breteau index and pupae per person) are reported here; correlated indices (container index, house index, and pupae per house) are listed in Supplemental Table 2.

#### Breteau index.

**Figure 2.**
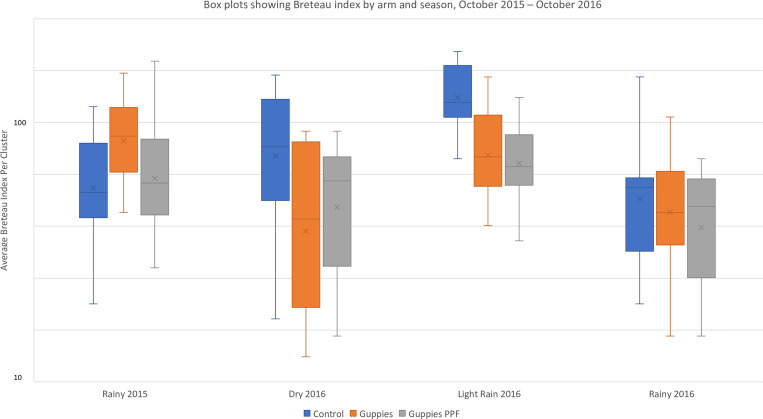
Box plots showing Breteau index (i.e., the number of containers positive for *Aedes* larvae and/or pupae per 100 houses surveyed) by arm and season, October 2015 to October 2016. This figure appears in color at www.ajtmh.org.

During the intervention period, the Breteau index was significantly less in both arm 1 (guppies, PPF, and COMBI) (AR = 0.64; 95% CI, 0.50–0.85; *P* = 0.0016) and arm 2 (guppies and COMBI) (AR = 0.63; 95% CI, 0.48–0.82; *P* = 0.0006) relative to arm 3 (control). The difference between the two intervention arms was not significant (AR = 0.97; 95% CI, 0.73–1.27; *P* = 0.7982) (Table 4). The biggest difference between arms was seen during the dry and light-rain or rainy seasons (Figure 2).

**Table 4:** Immature *Aedes* indices per cluster by arm and survey

	Breateau index		
	Control	Guppies	Guppies + PPF
Season			
Baseline (range)	62 (20–115)	91 (45–155)	69 (28–173)
Dry season (range)	88 (18–153)	48 (13–93)	54 (15–93)
Light rain (range)	130 (73–188)	81 (40–150)	74 (35–125)
Heavy rain (range)	58 (20–150)	51 (15–105)	45 (15–73)
Total (range)	92 (18–188)	60 (13–150)	58 (15–125)
Abundance ratio			
Abundance ratio (95% CI), *P* value*	1 (ref)	0.65 (0.50–0.85), *P* = 0.0016	0.63 (0.48–0.82), *P* = 0.0006
Abundance ratio (95% CI), *P* value*	†	1 (ref)	0.97 (0.73–1.27), *P* = 0.7982
	Pupae per person		
	Control	Guppies	Guppies + PPF
Season			
Baseline (range)	0.9 (0.2–2.7)	4.0 (0.2–17.1)	1.1 (0.5–2.3)
Dry season (range)	1.0 (0.1–3.3)	0.3 (0–0.9)	0.7 (0–1.7)
Light rain (range)	2.2 (0.5–7.0)	1.2 (0.1–3.3)	0.60 (0–1.4)
Heavy rain (range)	0.7 (0.1–2.1)	0.6 (0.1–2.9)	0.7 (0–1.8)
Total (range)	1.3 (0–7.0)	0.7 (0–3.3)	0.7 (0–1.8)
Abundance ratio			
Abundance ratio (95% CI), *P* value*	1 (ref)	0.56 (0.35–0.91), *P* = 0.0193	0.52 (0.32–0.84), *P* = 0.0075
Abundance ratio (95% CI), *P* value*	†	1 (ref)	0.92 (0.60–1.49), *P* = 0.7385

PPF = pyriproxyfen.

* The ratios do not include the baseline data.

† The ratio is not given here because it would be redundant.

#### Pupae per person.

**Figure 3.**
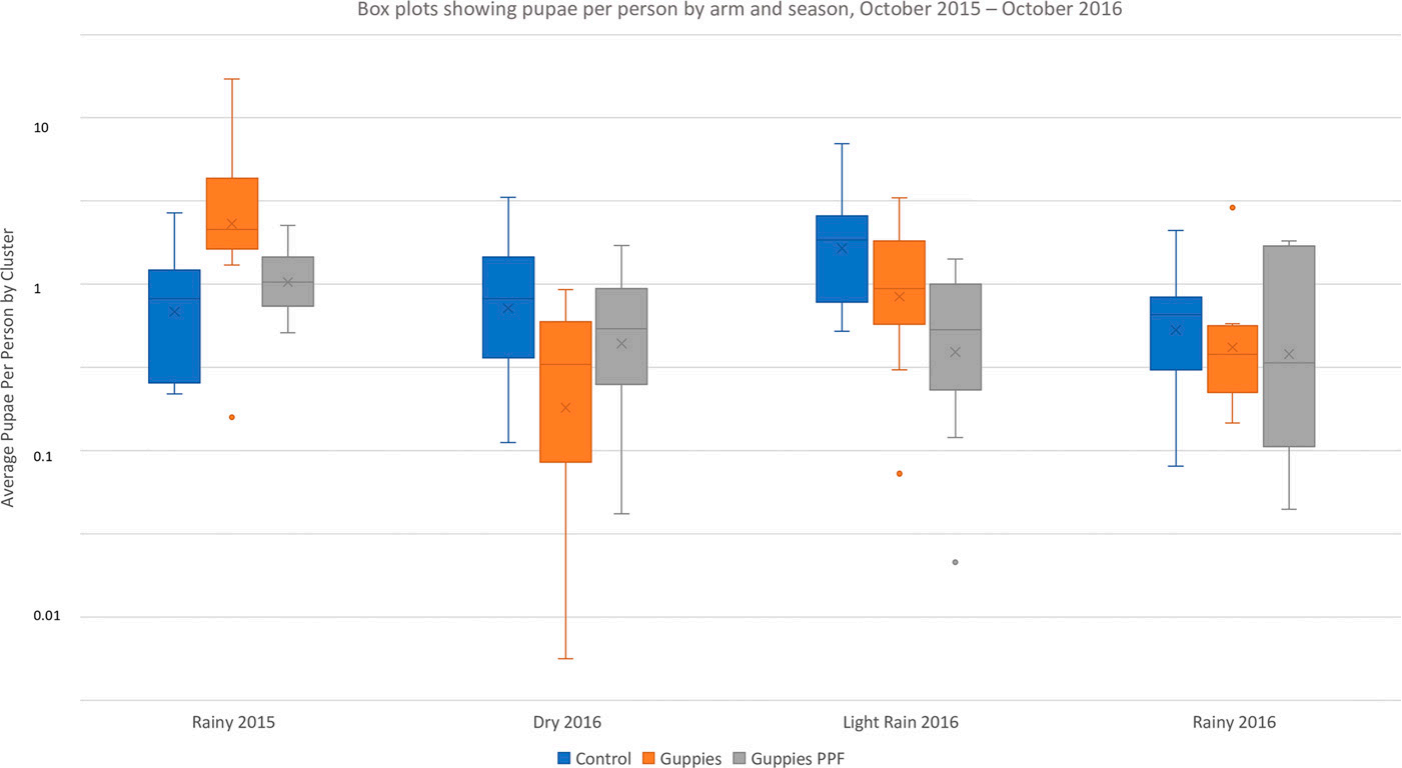
Box plots showing pupae per person (i.e., the number of *Aedes* pupae collected per person residing in the surveyed homes) by arm and season, October 2015 to October 2016. This figure appears in color at www.ajtmh.org.

Baseline results show significantly greater pupae per person in arm 2 (guppies and COMBI) than the other arms (Figure 3). During the intervention period, the number of pupae per person was significantly less in both arm 1 (guppies, PPF, and COMBI) (AR = 0.56; 95% CI, 0.35–0.91; *P* = 0.0193) and arm 2 (guppies and COMBI) (AR = 0.52; 95% CI, 0.32–0.84; *P* = 0.0075) relative to arm 3 (control). The difference between the two intervention arms was not significant (AR = 0.92; 95% CI, 0.60–1.49; *P* = 0.7385) (Table 4).

#### KAP survey.

The secondary outcome related to the KAP survey is reported here; more detail is included in the Supplemental Table 2. Other data from this KAP survey can be found in a previous publication.^48^ High levels of knowledge that dengue is transmitted by *Aedes* mosquitoes were reported at baseline among all arms (range, 95.5–98%). End-line surveys showed 100% of participants had this knowledge. Ratios of increased knowledge between baseline and end-line were not significantly different between arm 1 (guppies, PPF, and COMBI) (relative risk = 0.99; 95% CI, 0.86–0.1.14; *P* = 0.915) and arm 2 (guppies and COMBI) (relative risk = 1.01; 95% CI, 0.87–1.16; *P* = 0.943) relative to arm 3 (control) (Supplemental Table 2).

#### Coverage of guppy fish and Sumilarv 2MR.

**Figure 4.**
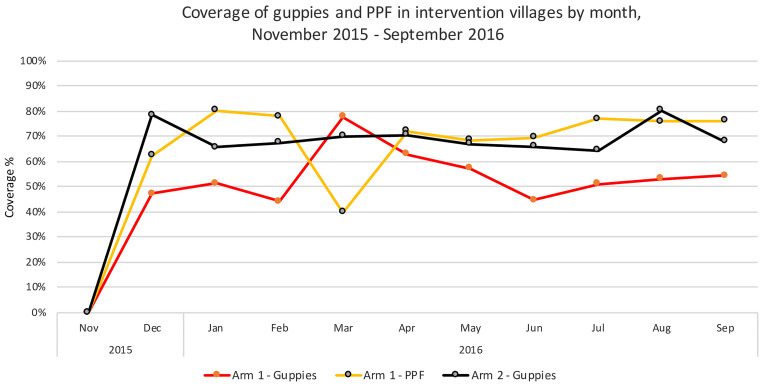
Coverage of guppies (i.e., proportion of eligible water containers with ≥ 1 guppy) and pyriproxyfen (i.e., proportion of eligible water containers with ≥ 1 Sumilarv^®^ MR) in intervention villages by month, November 2015 to September 2016. Arm 1 - Guppies in containers > 50 L; Arm 1 - PPF in containers 10–50 L; Arm 2 - Guppies in containers 10–50 L. This figure appears in color at www.ajtmh.org.

Coverage of guppy fish (proportion of eligible water containers with ≥ 1 guppy fish) before replacement in arm 2 increased to nearly 80% after 1 month and stayed close to 70% for most of the intervention period (Figure 4). However, in arm 1, PPF coverage (proportion of eligible water containers with ≥ 1 Sumilarv*^®^* MR) increased to 80% after 2 months and stayed high until decreasing in March, after which continued health education messages increased coverage back to near 70% to 80%. Guppy coverage in arm 1 was notably less (near 50%) until guppy use was emphasized in March, after which it increased dramatically and then dropped off back to around 50%.

#### Climate.

The average maximum daily temperature in the shade decreased from 34.4°C in the dry season to 31.3°C in the light-rain season. The average relative daily humidity and monthly rainfall increased from 60.0% and 10.7 mm to 75.2% and 139 mm from the dry to light-rain seasons, respectively (Supplement Figure 2). The rainy season saw much greater amounts of rainfall (near 300 mm per month) than all other seasons.

### Adverse events.

No adverse events, harm, or unintended effects were recorded during the trial.

## DISCUSSION

Guppies, regardless of whether they were in combination with PPF, were able to decrease the number of *Aedes* females (AR = 0.49—0.54) and pupae per person (AR = 0.52–0.56) by roughly half compared with the control, and resulted in approximately 35% decrease in the Breteau index (AR = 0.63–0.64). All other entomological indices also showed similar and statistically significant reductions in intervention arms compared with the control. Indeed, this is less than the 75% reduction that we posited in the sample size calculation, and the absolute values in all the arms are higher. The larval indices and adult indices were more highly correlated than usual.^49^ There were no statistical differences identified between the two intervention arms; however, it should be noted that the trial was not powered to detect these differences. Regardless, the lack of difference between the arms could also be a result of coverage. Guppy coverage was much less in intervention arm 1 (guppies, PPF, and COMBI) than arm 2 (guppies and COMBI) (≈50% versus ≈80%), suggesting the use of PPF may have contributed to keeping entomological indicators similar to those in arm 2.

It was observed that, during the heavy-rain season, the water containers often overflowed when raining and therefore caused larvae and pupae to flow out of the containers and die on the ground.^50^ This is one possible explanation for the marked decrease in the number of mosquitoes found in the heavy-rain season compared with the light-rain season. The abundance data presented here also follows the seasonality of *Aedes* mosquitoes described in other studies in the region.^51^ The interventions continued being replenished until the end of the trial.

Although none of the mosquito pools were found to be positive for dengue virus, all the positive and negative controls performed as expected. In addition, a model used to simulate the process of mosquito sampling, pooling, and virus testing found that mosquito infection rates commonly underestimate the prevalence of arbovirus infection in mosquito populations.^52^ This suggests that in our trial 1) the minimum infection rate found was the true rate in the population, 2) there was some degradation of RNA that resulted in untrue rates (despite proper cold chain management), or 3) the amount of virus in the pools was less than the detection threshold. In the future, studies investigators should try to acquire the largest number of mosquitoes as possible to identify the virus.

It was observed that adherence to guppies was high (70–80%) and consistent when only one intervention requiring behavior change (guppies) was assigned. In the intervention arm with guppies and PPF, adherence to one intervention was greatest when focused health education messages were provided for that intervention specifically (e.g., guppy coverage in March was greatest when guppy use was emphasized and was lowest in December to February when PPF use was emphasized). Similar dynamics have been found with the use of other vector control tools. A recent review concluded that, when applied as a single intervention, temephos was found to be effective at suppressing entomological indices. However, the same effect was not present when applied in combination with other interventions.^53^ This suggests that using multiple interventions that require behavior change may reduce individual intervention effectiveness. This presents a dilemma because single interventions may not be sufficient, but combining interventions may not improve efficacy either. Some studies have suggested that combining imperfect vector control with an imperfect medium-high efficacy vaccine could be a more efficacious and cost-effective way to reduce dengue cases.^54^^,^^55^ In addition, when selecting tools, control programs should consider the household coverage required to reach efficacy. For example, models have predicted a 70% to 75% coverage of targeted indoor residual sprays resulted in a 79% to 97% reduction in the number of dengue infections.^56^^,^^57^ The predicted efficacy for targeted indoor residual sprays is greater than what was reported using larvivorous fish in our study; however, these sprays may be more expensive, less community based, and more difficult to implement.

The results of the KAP survey showed very high knowledge levels, which may have resulted from the high number of cases in the study site and from previous government-led anti-dengue efforts in these areas.^48^ In addition, self-reported vector control practices did not match observed practices recorded in the surveys, nor was a correlation found between knowledge and observed practices.^48^ Therefore, an education campaign regarding dengue prevention in this setting with high knowledge levels is unlikely to have any significant effect on practices unless it is incorporated in a more comprehensive strategy for behavioral change (e.g., use of the COMBI method). To bridge the knowledge–practice gap, there is a need to create an enabling environment at the household, community, and health facility levels, and to have a continuous supply of the recommended interventions.

In previously reported FGDs and in-depth interviews, nearly all participants perceived that the interventions resulted in a reduction in *Aedes* mosquitos (both adults and immatures) and dengue cases.^50^ Participants showed high demand for both interventions (guppies and PPF) and were willing to pay between 100 to 500 riel (0.03–0.13 USD). In addition, several participants began rearing guppies in their home for their personal use, for the children to play with, and possibly to sell in the market. The major effect of PPF on mosquitoes is the inhibition of metamorphosis to prevent the emergence of adults from pupae.^20^ Therefore, the containers with PPF still had the presence of larvae in the water. This presence, despite the use of PPF, was a source of concern for some participants. Additional concerns reported were that children took the PPF from the containers to play with, and they were sometimes lost or misplaced when cleaning or replacing water. Overall, these issues were overcome in most cases with proper health education through health volunteers. Interpersonal communication through health volunteers was the most preferred method of transmitting prevention messages. Together, the entomological, KAP, and qualitative results suggest that the interventions were efficacious and accepted by the community.

However, there is always a need to balance potential benefits and harms of any intervention. Following the recent Zika outbreaks in 2015 and 2016, there were two groups of ecologists that noticed public health authorities using non-native larvivorous fish (including guppies) in *Aedes* control.^58^^,^^59^ Both of these groups wrote opinion pieces that gave three strong messages: 1) the use of larvivorous fish in vector control is not effective, 2) the chances of accidental guppy introduction into local ecosystems are large, and 3) guppies can establish populations easily and damage these aquatic ecosystems. The first point is contradicted by studies that were available at the time, as well as by the current trial.^17^^,^^19^^,^^28^ However, regarding the other points, guppies are indeed known to be highly plastic and acclimate to new environments.^60^ For example, as far back as 1963, guppies have been highly effective in *Culex* control in highly polluted ground pools and waterways in Bangkok, Yangon, and Taipei.^61^ In one study it was postulated that female guppies are capable of routinely establishing new populations in mesocosms, and that more than 80% of these populations persist for at least 2 years.^62^ Therefore, the key question is what is the ecological impact of guppies being released accidentally into the environment? Despite the strong statements made in the opinion pieces, the underlying evidence seems to be weaker than implied, with most introductions made before proper baseline assessments were completed. Studies have shown some effects of guppies on resident fish densities in laboratory conditions^63^^,^^64^ and nitrogen levels in water^65^^–^^67^; however, the extent of these effects across the ecosystem—especially in areas where introduction and naturalization took place many decades ago (such as Cambodia)— are far from settled. A book on evolutionary ecology of the guppy noted that, in regard to the impact of exotic guppies, “the literature is scant, and the area ripe for research.”^60^ That document author also noted that the manner in which introduced fish species affect native assemblages is incompletely understood, and that issues such as anthropogenic changes to the habitat, such as increases in water temperature, could favor introduced over native species.^60^

Measures available to control programs to mitigate the risks of introduction include 1) restricting breeding sites to areas that can be locked and controlled by the breeders, 2) only distributing fish to key containers in at-risk areas and away from lakes and streams, 3) only distributing male fish to avoid breeding after accidental release by households, and 4) evaluating which indigenous larvivorous fish exist that have similar predation behaviors to guppies and consider their use. It should be noted that male guppies have been found to consume less larvae than males (123 larvae/day compared with 74 larvae/day).^19^ However, this consumption rate was more than enough to clear the main breeding jars in Cambodia.

In addition to concerns about accidental release of guppies to the environment, some laboratory experiments have raised the possibility that putting guppies in containers used for drinking water could increase *Escherichia coli* and other bacteria.^68^ However, a recent study found the addition of guppy fish in Lao and Cambodia made no significant difference to high preexisting baseline levels of contamination (Jeffrey Hii, personal communication). Therefore, the authors concluded that any contaminating effect may be insignificant when compared with the potential for reducing dengue fever cases, and they advocated for the inclusion of advice on safe water use to be included in any behavior change communication programs for guppy introduction.

Our study has several limitations. The most important of which is the absence of a primary outcome related directly to dengue incidence, rather than an entomological one. Finding the appropriate metric to measure disease impact is bedeviled by the effect of human movement on patterns of transmission, and the pronounced temporal and spatial heterogeneity in transmission, which necessitate very large cluster-randomized study designs.^69^^,^^70^ We considered passive surveillance for dengue with rapid diagnostic tests in HCs. Although sensitivity among currently available tests was considered acceptable for routine clinical diagnostics,^71^ it was not considered high enough for seroconversion studies, and no studies were identified that had used rapid diagnostics to estimate seroprevalence. Therefore, more expensive and labor-intensive efforts were preferable, such as cohort studies or capture–recapture methods (which have their own limitations^72^) to estimate the true number of cases, or using a more sensitive diagnostic tool such as reverse transcription–polymerase chain reaction. However, because of budget limitations, it was not possible to use them. In addition, unpublished data from a recent cohort study in the proposed districts suggest that, given a similar number of cases during this study time frame, and the resources available to the current project, there would not be enough statistical power to show an impact of the likely size on case numbers.^31^ Therefore, the endpoint chosen was the abundance of adult *Aedes* mosquitoes, which are on the causal pathway to disease.

Nevertheless, determining the effect of an entomological outcome on dengue transmission is difficult. Multiple studies in Cuba have suggested that a Breteau index > 5 can be used to predict dengue transmission, although they note that their results can probably not be extrapolated to areas were dengue transmission is endemic.^73^^,^^74^ A recent study from Peru did show a statistically significant association between 12-month longitudinal data on *Aedes aegypti* abundance (1.01–1.30) and categorical immature indices (1.21–1.75) on risk ratios dengue virus seroconversion (over 5 months).^73^ However, the existence of an association remains less certain across different geographies, and what the strength of that association would be in Cambodia (with much greater incidence rates) remains difficult to quantify. These efforts are frustrated by the many intersecting factors that determine dengue infection in communities, including the probability of infecting and being infected by a mosquito bite, the duration of infection, treatment-seeking behavior, the risk of fever, which serotypes are present, acquired immunity in the host, coverage of interventions, and background prevalence of dengue infections. The availability of quality data for each of these factors is limited in most tropical countries where the infection rates are highest.

Additional entomological limitations include only having one data collection point in each season, and no measure in the change of parity rate of adult females. The indoor resting collection of *Aedes* adult mosquitoes is subject to many challenges including 1) individual collector performance and efficiency, 2) abundance being time dependent, and 3) housing conditions, architecture, objects, and so on. Possible ways to improve collector performance and efficiency could include using electronic data collection, which can record the time and place of each entry, allowing easier monitoring and supervision of collection teams and the reduction of data transcription errors. Electronic timers can be used to ensure each collector aspirates for the proper time. Investigators can also look to the WHO Vector Control Advisory Group for their recent guidance on how to design vector control efficacy trials.^75^ Another possible source of bias is not having data collectors blind to the intervention; however, in our case it was unavoidable because data collection teams were able to see the fish in the containers they sampled. In addition, because these data were collected within one province in Cambodia, generalizability could be a concern. However, it is likely that the result of this trial could be generalizable to areas with similar ecology and mosquito densities within the country and in neighboring countries. In addition, research in Vietnam found a narrow range of critical human population densities between around 3,000 to 7,000 people/km^2^ prone to dengue outbreaks and concluded that rural areas may contribute at least as much to the dissemination of dengue fever as cities.^76^

In addition to the limitations described, resistance can impair the effectiveness of vector control interventions. Even insecticidal interventions shown to be highly effective in some areas, such as targeted indoor residual spraying,^77^ depend on the resistance status of the mosquitoes in the area.^78^ Although larvivorous fish may be effective in some settings, they likely will not work in all settings, especially in urban areas or other setting in which the key containers or breeding areas are not suitable for fish.

In conclusion, the results from this trial indicate that the interventions resulted in a statistically significant reduction in immature and adult *Aedes* mosquito abundance when compared with the control. There were no statistical differences identified between intervention arms, although lower guppy coverage in intervention arm 2 suggests that PPF did help keep mosquito densities low. Data from the KAP surveys and qualitative assessments showed that the interventions were accepted by communities and that they were willing to pay for them. The extremely low cost of including guppy rearing in community-based health structures along with the effectiveness demonstrated here suggest guppies should be considered as a vector control tool as long as the benefits outweigh any potential environmental concerns. PPF was also highly accepted and preferred over current vector control tools used in Cambodia; however, product costs and availability are still unknown. The qualitative assessment suggests that a context-specific and well-informed COMBI and community engagement by giving an active role to communities is the key to the successful dengue control. Additional studies could be done to confirm these results and explore the effect of the interventions in different ecological conditions.

## Supplemental Material


Supplemental materials

## References

[1] Seng CM Setha T Nealon J Socheat D Nathan MB, 2008. Six months of *Aedes aegypti* control with a novel controlled-release formulation of pyriproxyfen in domestic water storage containers in Cambodia. Southeast Asian J Trop Med Public Health 39: 822–826. 19058575

[2] Vannice KS Roehrig JT Hombach J, 2015. Next generation dengue vaccines: a review of the preclinical development pipeline. Vaccine 33: 7091–7099. 26424602 10.1016/j.vaccine.2015.09.053

[3] Alphey L Mckemey A Nimmo D Oviedo MN Lacroix R Matzen K Beech C, 2013. Genetic control of *Aedes* mosquitoes. Pathog Glob Health 107: 170–179. 23816508 10.1179/2047773213Y.0000000095 PMC4001467

[4] Franz AWE Clem RJ Passarelli AL, 2014. Novel genetic and molecular tools for the investigation and control of dengue virus transmission by mosquitoes. Curr Trop Med Rep 1: 21–31. 24693489 10.1007/s40475-013-0007-2 PMC3969738

[5] Ye YH Carrasco AM Frentiu FD Chenoweth SF Beebe NW van den Hurk AF Simmons CP O’Neill SL McGraw EA, 2015. *Wolbachia* reduces the transmission potential of dengue-infected *Aedes aegypti.* PLoS Negl Trop Dis. Available at: 10.1371/journal.pntd.0003894. PMC4482661 26115104

[6] Turelli M Barton NH, 2017. Deploying dengue-suppressing *Wolbachia*: robust models predict slow but effective spatial spread in *Aedes aegypti.* Theor Popul Biol 115: 45–60. 28411063 10.1016/j.tpb.2017.03.003 PMC5476474

[7] Bowman LR Donegan S McCall PJ, 2016. Is dengue vector control deficient in effectiveness or evidence? Systematic review and meta-analysis. PLoS Negl Trop Dis 10: e0004551. 26986468 10.1371/journal.pntd.0004551 PMC4795802

[8] Huy R , 2010. National dengue surveillance in Cambodia 1980–2008: epidemiological and virological trends and the impact of vector control. Bull World Health Organ 88: 650–657. 20865069 10.2471/BLT.09.073908 PMC2930366

[9] Vong S Goyet S Ly S Ngan C Huy R Duong V Wichmann O Letson GW Margolis HS Buchy P, 2012. Under-recognition and reporting of dengue in Cambodia: a capture–recapture analysis of the National Dengue Surveillance System. Epidemiol Infect 140: 491–499. 21733251 10.1017/S0950268811001191

[10] Wichmann O , 2011. Dengue in Thailand and Cambodia: an assessment of the degree of underrecognized disease burden based on reported cases. PLoS Negl Trop Dis. Available at: 10.1371/journal.pntd.0000996. PMC3066139 21468308

[11] Seng CM Setha T Nealon J Chantha N Socheat D Nathan MB, 2008. The effect of long-lasting insecticidal water container covers on field populations of *Aedes aegypti* (L.) mosquitoes in Cambodia. J Vector Ecol 33: 333–341. 19263854 10.3376/1081-1710-33.2.333

[12] Kuhn S Manderson LH, 2007. Abate distribution and dengue control in rural Cambodia. Acta Trop 101: 139–146. 17291439 10.1016/j.actatropica.2007.01.002

[13] Boyer S , 2018. Resistance of *Aedes aegypti* (Diptera: Culicidae) populations to deltamethrin, permethrin, and temephos in Cambodia. Asia-Pacific J Public Health 30: 158–166. 10.1177/1010539517753876 29502428

[14] Polson KA Curtis C Seng CM Olson JG Chantha N Rawlins SC, 2001. Susceptibility of two Cambodian population of *Aedes aegypti* mosquito larvae to temephos during 2001. Dengue Bull 25: 79–83.

[15] Seng CM Setha T Chanta N Socheat D Guillet P Nathan MB, 2006. Inhibition of adult emergence of *Aedes aegypti* in simulated domestic water-storage containers by using a controlled-release formulation of pyriproxyfen. J Am Mosq Control Assoc 22: 152–154. 16646342 10.2987/8756-971X(2006)22[152:IOAEOA]2.0.CO;2

[16] Setha T Chantha N Socheat D, 2007. Efficacy of *Bacillus thuringiensis israelensis*, VectoBac WG and DT, formulations against dengue mosquito vectors in cement potable water jars in Cambodia. Southeast Asian J Trop Med Public Health 38: 261–268. 17539275

[17] World Health Organization , 2013. *Managing Regional Public Goods for Health: Community-based Dengue Vector Control*. Available at: https://www.adb.org/sites/default/files/publication/30167/community-based-dengue-vector-control.pdf. Accessed July 19, 2021.

[18] Congpuong K Bualombai P Banmairuroi V Na-Bangchang K, 2010. Compliance with a three-day course of artesunate–mefloquine combination and baseline anti-malarial treatment in an area of Thailand with highly multidrug resistant falciparum malaria. Malar J 9: 43. 20132537 10.1186/1475-2875-9-43 PMC2829592

[19] Seng C Setha T Nealson J Socheat D Chantha N Nathan M, 2008. Community-based use of larvivorous fish *Pocecilia reticulata* to control the dengue vector *Aedes aegypti* in domestic water storage containers in rural Cambodia. J Vector Ecol 33: 139–144. 18697316 10.3376/1081-1710(2008)33[139:cuotlf]2.0.co;2

[20] World Health Organization , 2000. *Report of the Fourth WHOPES Working Group Meeting: review of IR3535, KBR3023, (RS)-Methoprene 20% EC and Pyriproxyfen 0.5% GR.* Available at: https://apps.who.int/iris/handle/10665/66683.

[21] Sai-Zaw-Min-Oo, Sein-Thaung, Yan-Naung-Maung-Maung, Khin-Myo-Aye, Zar-Zar-Aung, Hlaing-Myat-Thu, Kyaw-Zin-Thant, Minakawa N, 2018. Effectiveness of a novel long-lasting pyriproxyfen larvicide (SumiLarv^®^2MR) against *Aedes* mosquitoes in schools in Yangon, Myanmar. Parasit Vectors 11: 16. 29306333 10.1186/s13071-017-2603-9 PMC5756364

[22] Suaya JA Shepard DS Chang MS Caram M Hoyer S Socheat D Chantha N Nathan MB, 2007. Cost-effectiveness of annual targeted larviciding campaigns in Cambodia against the dengue vector *Aedes aegypti.* Trop Med Int Health 12: 1026–1036. 17875014 10.1111/j.1365-3156.2007.01889.x

[23] Azmawati MN Aniza I Ali M, 2013. Evaluation of communication for behavioral impact (COMBI) program in dengue prevention: a qualitative and quantitative study in Selangor, Malaysia. Iran J Public Health 42: 538–539. 23802114 PMC3684465

[24] Al-Muhandis N Hunter PR, 2011. The value of educational messages embedded in a community-based approach to combat dengue fever: a systematic review and meta regression analysis. PLoS Negl Trop Dis 5: e1278. 21886848 10.1371/journal.pntd.0001278 PMC3160295

[25] Heintze C Velasco Garrido M Kroeger A, 2007. What do community-based dengue control programmes achieve? A systematic review of published evaluations. Trans R Soc Trop Med Hyg 101: 317–325. 17084427 10.1016/j.trstmh.2006.08.007

[26] Vanlerberghe V Toledo ME Rodriguez M Gomez D Baly A Benitez JR Van der Stuyft P, 2010. Community involvement in dengue vector control: cluster randomised trial. MEDICC Rev 12: 41–47. 20387334

[27] Castro M Sanchez L Perez D Carbonell N Lefevre P Vanlerberghe V Van der Stuyft P, 2012. A community empowerment strategy embedded in a routine dengue vector control programme: a cluster randomised controlled trial. Trans R Soc Trop Med Hyg 106: 315–321. 22465423 10.1016/j.trstmh.2012.01.013

[28] Han WW Lazaro A McCall PJ George L Runge-Ranzinger S Toledo J Velayudhan R Horstick O, 2015. Efficacy and community effectiveness of larvivorous fish for dengue vector control. Trop Med Int Health 20: 1239–1256. 25962851 10.1111/tmi.12538

[29] Campbell MK Piaggio G Elbourne DR Altman DG, 2012. Consort 2010 statement: Extension to cluster randomised trials. BMJ 345: e5661. 22951546 10.1136/bmj.e5661

[30] Harrington LC , 2005. Dispersal of the dengue vector *Aedes aegypti* within and between rural communities. Am J Trop Med Hyg 72: 209–220. 15741559

[31] Hustedt J , 2017. Determining the efficacy of guppies and pyriproxyfen (Sumilarv^®^ 2MR) combined with community engagement on dengue vectors in Cambodia: study protocol for a randomized controlled trial. Trials 18: 367. 28778174 10.1186/s13063-017-2105-2 PMC5545006

[32] World Health Organization , 2017. *WHO Specifications and Evaluations for Public Health Pesticides: Pyriproxyfen*. Available at: https://www.who.int/whopes/quality/Pyriproxyfen_eval_specs_WHO_July_2017.pdf. Accessed July 19, 2021.

[33] World Health Organization , 2017. *Report of the Twentieth WHOPES Working Group Meeting*. https://apps.who.int/iris/bitstream/handle/10665/258921/WHO-HTM-NTD-WHOPES-2017.04-eng.pdf. Accessed July 19, 2021.

[34] Hemming KMJ, 2013. A menu-driven facility for sample-size calculations in cluster randomized controlled trials. Stata J 13: 114–135.

[35] Hayes RJ Alexander ND Bennett S Cousens SN, 2000. Design and analysis issues in cluster-randomized trials of interventions against infectious diseases. Stat Methods Med Res 9: 95–116. 10946429 10.1177/096228020000900203

[36] Khun S, Manderson L, 2007. Community and school-based health education for dengue control in rural Cambodia: a process evaluation. *PLoS Negl Trop Dis* *1:* e143. 10.1371/journal.pntd.0000143 PMC2154392 18160981

[37] Rowland M , 2004. DEET mosquito repellent provides personal protection against malaria: a household randomized trial in an Afghan refugee camp in Pakistan. Trop Med Int Health 9: 335–342. 14996362 10.1111/j.1365-3156.2004.01198.x

[38] Campbell MK Fayers PM Grimshaw JM, 2005. Determinants of the intracluster correlation coefficient in cluster randomized trials: the case of implementation research. Clin Trials 2: 99–107. 16279131 10.1191/1740774505cn071oa

[39] World Health Organization , 2009. Dengue: Guidelines for Diagnosis, Treatment, Prevention, and Control. Spec Program Res Train Trop Dis. 23762963

[40] Tun-Lin W Kay BH Barnes A, 1995. The premise condition index: a tool for streamlining surveys of *Aedes aegypti.* Am J Trop Med Hyg 53: 591–594. 8561259 10.4269/ajtmh.1995.53.591

[41] Hustedt J , 2020. Ability of the premise condition index to identify premises with adult and immature *Aedes* mosquitoes in Kampong Cham, Cambodia. *Am J Trop Med Hyg* 102: 1432–1439. 32274992 10.4269/ajtmh.19-0453 PMC7253129

[42] Sule WF Oluwayelu DO, 2016. Analysis of *Culex* and *Aedes* mosquitoes in southwestern Nigeria revealed no West Nile virus activity. Pan Afr Med J 23: 116. 27279943 10.11604/pamj.2016.23.116.7249 PMC4885691

[43] Cevallos V Ponce P Waggoner JJ Pinsky BA Coloma J Quiroga C Morales D Cárdenas MJ, 2018. Zika and Chikungunya virus detection in naturally infected *Aedes aegypti* in Ecuador. Acta Trop 177: 74–80. 28982578 10.1016/j.actatropica.2017.09.029

[44] Pierre V Drouet MT Deubel V, 1994. Identification of mosquito-borne flavivirus sequences using universal primers and reverse transcription/polymerase chain reaction. Res Virol 145: 93–104. 7520190 10.1016/s0923-2516(07)80011-2

[45] Lanciotti RS Calisher CH Gubler DJ Chang GJ Vorndam AV, 1992. Rapid detection and typing of dengue viruses from clinical samples by using reverse transcriptase-polymerase chain reaction. J Clin Microbiol 30: 545–551. 1372617 10.1128/jcm.30.3.545-551.1992 PMC265106

[46] Grant AM , 2005. Issues in data monitoring and interim analysis of trials. Heal Technol Assess 9: 1–238, iii–iv. 10.3310/hta9070 15763038

[47] Association AA , 2012. *Principles of Professional Responsibility*. Available at: http://ethics.aaanet.org/category/statement/.

[48] Kumaran E , 2018. Dengue knowledge, attitudes and practices and their impact on community-based vector control in rural Cambodia. PLoS Negl Trop Dis 12: e0006268. 29451879 10.1371/journal.pntd.0006268 PMC5833285

[49] Vanlerberghe V Toledo ME Rodriguez M Gomez D Baly A Benitez JR Van der Stuyft P, 2009. Community involvement in dengue vector control: cluster randomised trial. BMJ 338: b1959. 19509031 10.1136/bmj.b1959 PMC2694260

[50] Shafique M , 2019. Implementation of guppy fish (*Poecilia reticulata*), and a novel larvicide (Pyriproxyfen) product (Sumilarv 2MR) for dengue control in Cambodia: a qualitative study of acceptability, sustainability and community engagement. PLoS Negl Trop Dis 13: e0007907. 31738759 10.1371/journal.pntd.0007907 PMC6886868

[51] Lamaningao P Lamaningao P Kanda S Kanda S Shimono T Shimono T Inthavongsack S Xaypangna T Nishiyama T, 2020. *Aedes* mosquito surveillance and the use of a larvicide for vector control in a rural area of the Lao People’s Democratic Republic. *Trop Med Health* 48: 54. 32612446 10.1186/s41182-020-00242-7 PMC7325043

[52] Bustamante DM Lord CC, 2010. Sources of error in the estimation of mosquito infection rates used to assess risk of arbovirus transmission. Am J Trop Med Hyg 82: 1172–1184. 20519620 10.4269/ajtmh.2010.09-0323 PMC2877431

[53] George L Lenhart A Toledo J Lazaro A Han WW Velayudhan R Runge Ranzinger S Horstick O, 2015. Community-effectiveness of temephos for dengue vector control: a systematic literature review. PLoS Negl Trop Dis 9: e0004006. 26371470 10.1371/journal.pntd.0004006 PMC4570708

[54] Fitzpatrick C Haines A Bangert M Farlow A Hemingway J Velayudhan R, 2017. An economic evaluation of vector control in the age of a dengue vaccine. PLoS Negl Trop Dis 11: e0005785. 28806786 10.1371/journal.pntd.0005785 PMC5573582

[55] Christofferson RC Mores CN, 2015. A role for vector control in dengue vaccine programs. Vaccine 33: 7069–7074. 26478199 10.1016/j.vaccine.2015.09.114

[56] Hladish TJ Pearson CAB Rojas DP Gomez-Dantes H Halloran ME Vazquez-Prokopec GM Longini IM, 2018. Forecasting the effectiveness of indoor residual spraying for reducing dengue burden. PLoS Negl Trop Dis. Available at: 10.1371/journal.pntd.0006570. PMC6042783 29939983

[57] Cavany SM , 2020. Optimizing the deployment of ultra-low volume and targeted indoor residual spraying for dengue outbreak response. *PLOS Comput Biol.* Available at: 10.1371/journal.pcbi.1007743. PMC7200023 32310958

[58] El-Sabaawi RW Frauendorf TC Marques PS Mackenzie RA Manna LR Mazzoni R Phillip DAT Warbanski ML Zandonà E, 2016. Biodiversity and ecosystem risks arising from using guppies to control mosquitoes. *Biol Lett.* Available at: 10.1098/rsbl.2016.0590. PMC5095194 28120806

[59] Azevedo-Santos VM Vitule JRS Pelicice FM García-Berthou E Simberloff D, 2017. Nonnative fish to control *Aedes* mosquitoes: a controversial, harmful tool. Bioscience 67: 84–90.

[60] Magurran AE, 2005. Evolutionary Ecology: The Trinidadian Guppy. Oxford, UK: Oxford University Press.

[61] Bay EC Self LS, 1972. Observations of the guppy, *Poecilia reticulata* Peters, in *Culex pipiens fatigans* breeding sites in Bangkok, Rangoon, and Taipei. Bull World Health Organ 46: 407–416. 4261424 PMC2480748

[62] Deacon AE Ramnarine IW Magurran AE, 2011. How reproductive ecology contributes to the spread of a globally invasive fish. *PLoS One.* Available at: 10.1371/journal.pone.0024416. PMC3176282 21957449

[63] Walsh MR Reznick DN, 2010. Influence of the indirect effects of guppies on life-history evolution in *Rivulus hartii* . Evolution (NY). Available at: 10.1111/j.1558-5646.2009.00922.x. 20015237

[64] Walsh MR Reznick DN, 2011. Experimentally induced life-history evolution in a killifish in response to the introduction of guppies. Evolution (NY) 65: 1021–1036. 10.1111/j.1558-5646.2010.01188.x 21062280

[65] Holitzki TM MacKenzie RA Wiegner TN McDermid KJ, 2013. Differences in ecological structure, function, and native species abundance between native and invaded Hawaiian streams. Ecol Appl 23: 1367–1383. 24147409 10.1890/12-0529.1

[66] El-Sabaawi RW Marshall MC Bassar RD López-Sepulcre A Palkovacs EP Dalton C, 2015. Assessing the effects of guppy life history evolution on nutrient recycling: from experiments to the field. Freshw Biol 60: 590–601.

[67] Collins SM , 2016. Fish introductions and light modulate food web fluxes in tropical streams: a whole-ecosystem experimental approach. Ecology. Available at: 10.1002/ecy.1530. 27870030

[68] Chadee DD, 1992. Bacterial pathogens isolated from guppies (*Poecilia reticulata*) used to control *Aedes aegypti* in Trinidad. Trans R Soc Trop Med Hyg 86: 693. 1287947 10.1016/0035-9203(92)90194-h

[69] Van Breukelen GJP Candel MJJM, 2012. Calculating sample sizes for cluster randomized trials: we can keep it simple and efficient! J Clin Epidemiol 65: 1212-1218. 23017638 10.1016/j.jclinepi.2012.06.002

[70] Hayes RJ Moulton LH, 2009. Cluster Randomized Trials. Boca Raton, FL: Chapman & Hall/CRC Press.

[71] Hunsperger EA , 2014. Evaluation of commercially available diagnostic tests for the detection of dengue virus NS1 antigen and anti-dengue virus IgM antibody. PLoS Negl Trop Dis 8: e3171. 25330157 10.1371/journal.pntd.0003171 PMC4199549

[72] Tilling K, 2001. Capture–recapture methods: useful or misleading? Int J Epidemiol 30: 12–14. 11171841 10.1093/ije/30.1.12

[73] Sanchez L Vanlerberghe V Alfonso L Marquetti MDC Guzman MG Bisset J Van Der Stuyft P, 2006. *Aedes aegypti* larval indices and risk for dengue epidemics. Emerg Infect Dis 12: 800–806. 16704841 10.3201/eid1205.050866 PMC3374431

[74] Sanchez L Cortinas J Pelaez O Gutierrez H Concepción D Van Der Stuyft P, 2010. Breteau index threshold levels indicating risk for dengue transmission in areas with low *Aedes* infestation. *Trop Med Int Health* *15:* 173–175. 10.1111/j.1365-3156.2009.02437.x 20409286

[75] Vector Control Advisory Group

[76] Schmidt W-P , 2011. Population density, water supply, and the risk of dengue fever in Vietnam: cohort study and spatial analysis. PLoS Med. Available at: 10.1371/journal.pmed.1001082. PMC3168879 21918642

[77] Vazquez-Prokopec GM Montgomery BL Horne P Clennon JA Ritchie SA, 2017. Combining contact tracing with targeted indoor residual spraying significantly reduces dengue transmission. Sci Adv 3: e1602024. 28232955 10.1126/sciadv.1602024 PMC5315446

[78] Vazquez-Prokopec GM , 2017. Deltamethrin resistance in *Aedes aegypti* results in treatment failure in Merida, Mexico. PLoS Negl Trop Dis. Available at: 10.1371/journal.pntd.0005656. PMC5481028 28604781

