# Supplementary material for: Field Efficacy of Larvivorous Fish and Pyriproxyfen Combined with Community Engagement on Dengue Vectors in Cambodia: A Randomized Controlled Trial
Source: Am J Trop Med Hyg. 2021 Sep 7;105(5):1265–76. doi: 10.4269/ajtmh.20-1088 (PMC8592206; doi:10.4269/ajtmh.20-1088)
Supplement: Supplementary file 2 [file tpmd201088.SD2.pdf]

Supplemental Figure S1: CONSORT 2010 Flow Diagram for enrolment, follow up, and analysis of clusters

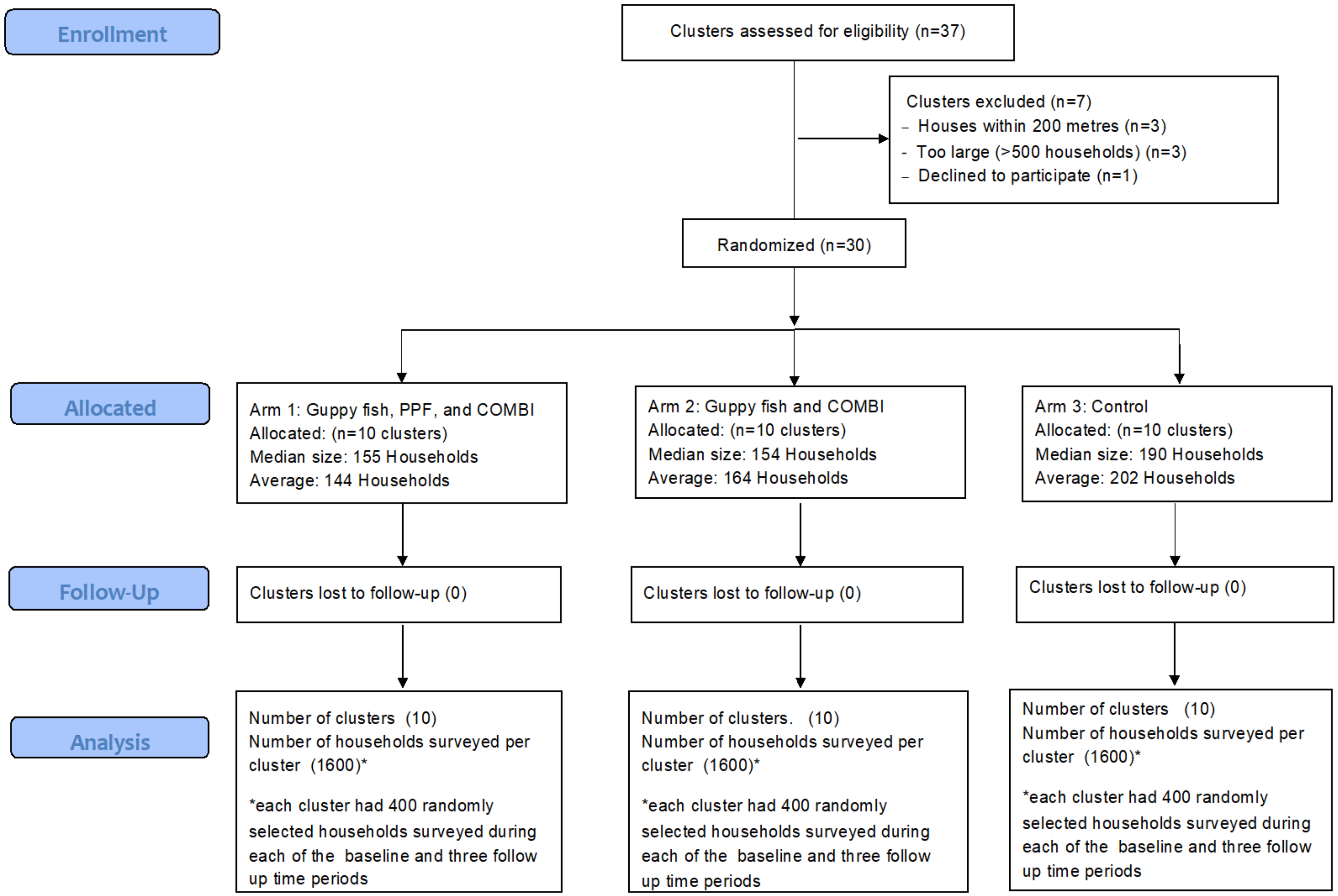

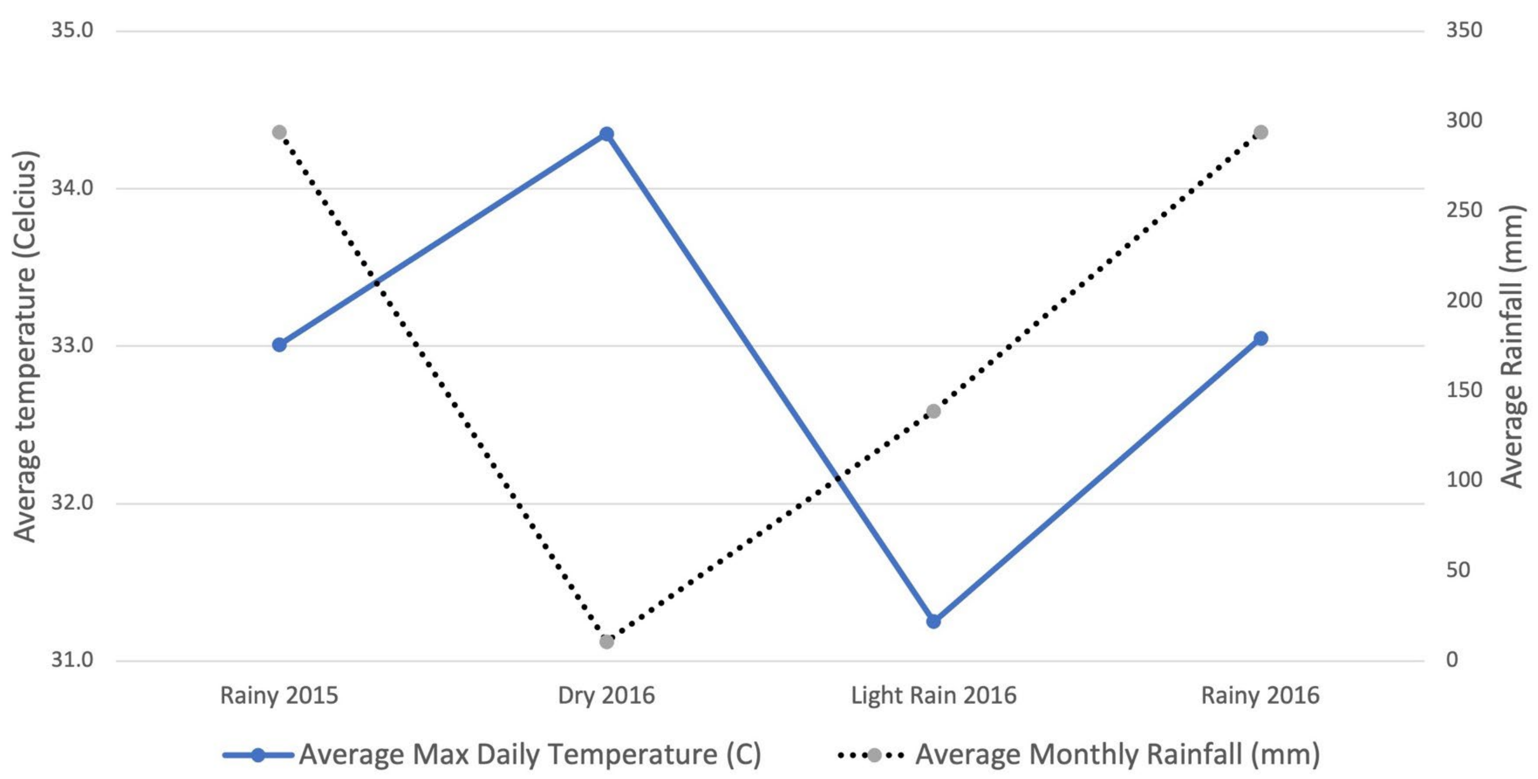

35.0
34.0
33.0
32.0
31.0
Average temperature (Celcius)
350
300
250
200
150
100
50
0
Average Rainfall (mm)
Rainy 2015
Dry 2016
Light Rain 2016
Rainy 2016
Average Max Daily Temperature (C)
Average Monthly Rainfall (mm)

cluster	survey	house	arm	_2house	_2yard	_2shade	_2water	pci3	pci4	aedes_fem	hhmember	pupae_wei	cont_total	cont_positi	house_pos	housеindex	containerir	pupaeperhouse
29	4	40	1	1.925	1.85	1.975	2	5.75	7.75	7	177	116	319	22	14	0.35	0.068966	2.9
30	2	40	1	2	2	1.3	2	5.3	7.3	8	165	257	357	30	14	0.35	0.084034	6.425
29	2	40	1	1.9	2.025	1.375	2	5.3	7.3	31	200	666	427	61	16	0.4	0.142857	16.65
24	3	40	1	1.9	1.975	1.775	2	5.65	7.65	56	189	1317	302	64	28	0.7	0.211921	32.925
26	2	40	1	1.975	1.925	1.725	2	5.625	7.625	14	210	298	417	46	18	0.45	0.110312	7.45
27	3	40	1	2.225	2	2.025	2	6.25	8.25	57	200	501	264	45	29	0.725	0.170455	12.525
21	4	40	1	1.975	1.925	1.8	2	5.7	7.7	11	154	39	229	26	16	0.4	0.113537	0.975
24	4	40	1	2	2.025	2.075	2.025	6.1	8.125	23	210	17	233	12	9	0.225	0.051502	0.425
29	3	40	1	1.9	1.975	2.125	2	6	8	134	192	100	260	57	30	0.75	0.219231	2.5
23	2	40	1	2.1	2.025	1.575	2	5.7	7.7	15	179	20	263	7	4	0.1	0.026616	0.5
30	3	40	1	2.15	2.125	1.925	2.025	6.2	8.225	64	163	450	191	75	38	0.95	0.39267	11.25
23	4	40	1	2.05	2.175	1.925	2	6.15	8.15	7	174	139	191	24	19	0.475	0.125654	3.475
25	3	40	1	2.05	1.95	2.05	2	6.05	8.05	181	181	380	275	44	27	0.675	0.16	9.5
23	3	40	1	2	1.575	1.6	2	5.175	7.175	17	188	156	204	36	23	0.575	0.176471	3.9
25	2	40	1	1.975	2	1.375	2	5.35	7.35	19	165	115	368	15	11	0.275	0.040761	2.875
30	4	40	1	1.9	1.875	2.2	2	5.975	7.975	15	169	355	323	60	26	0.65	0.185759	8.875
26	4	40	1	2.05	2	2	2	6.05	8.05	6	192	176	236	13	10	0.25	0.055085	4.4
27	2	40	1	2.3	2.05	1.675	2	6.025	8.025	36	189	181	412	45	20	0.5	0.109223	4.525
27	4	40	1	1.975	2	2	2	5.975	7.975	10	200	161	251	8	5	0.125	0.031873	4.025
26	3	40	1	2.15	2.1	1.85	2	6.1	8.1	74	164	352	180	51	29	0.725	0.283333	8.8
22	4	40	1	1.975	2.15	1.675	2	5.8	7.8	8	187	91	220	21	14	0.35	0.095455	2.275
24	2	40	1	2.051282	2.128205	1.384615	2	5.564103	7.564103	49	198	52	348	35	17	0.425	0.100575	1.3
21	3	40	1	2.025	1.9	1.825	2	5.75	7.75	37	155	100	291	29	18	0.45	0.099656	2.5
22	2	40	1	2.05	2.075	1.425	2	5.55	7.55	3	194	103	333	22	10	0.25	0.066066	2.575
22	3	40	1	2.125	1.65	1.675	2	5.45	7.45	46	211	306	212	45	24	0.6	0.212264	7.65
25	4	40	1	2	2.475	1.625	2	6.1	8.1	5	163	106	194	23	17	0.425	0.118557	2.65
28	3	40	1	2.25	1.975	1.95	2	6.175	8.175	86	160	260	249	74	36	0.9	0.297189	6.5
28	4	40	1	2.125	2.2	1.75	2.025	6.075	8.1	4	139	45	233	24	17	0.425	0.103004	1.125
28	2	40	1	1.974359	2.025641	1.815789	2	5.81579	7.81579	16	151	174	414	60	20	0.5	0.144928	4.35
21	2	40	1	2	2.025	1.225	2	5.25	7.25	6	137	55	281	29	12	0.3	0.103203	1.375
10	2	40	2	1.95	1.875	1.975	2	5.8	7.8	17	159	50	344	18	10	0.25	0.052326	1.25
14	4	40	2	1.975	2.2	1.725	2	5.9	7.9	2	158	88	227	23	15	0.375	0.101322	2.2
18	2	40	2	2.2	2.075	1.25	2	5.525	7.525	6	184	22	310	8	6	0.15	0.025806	0.55
7	4	40	2	1.975	1.75	2.225	2	5.95	7.95	23	195	104	252	42	24	0.6	0.166667	2.6
5	2	40	2	2.025	2	1.825	2	5.85	7.85	11	178	1	384	5	3	0.075	0.013021	0.025
14	3	40	2	1.925	2	1.975	2	5.9	7.9	71	189	311	282	60	20	0.5	0.212766	7.775
6	2	40	2	2	2	1.275	2	5.275	7.275	3	193	66	337	16	8	0.2	0.047478	1.65
1	4	40	2	1.95	1.725	2.075	2	5.75	7.75	8	189	39	230	14	8	0.2	0.06087	0.975
3	4	40	2	1.875	2.075	1.875	1.875	5.825	7.7	19	163	469	192	29	19	0.475	0.151042	11.725
9	2	40	2	1.975	1.975	1.1	2	5.05	7.05	10	192	6	373	7	5	0.125	0.018767	0.15
1	2	40	2	2.05	2	1.5	2	5.55	7.55	14	180	121	360	21	9	0.225	0.058333	3.025
7	3	40	2	2	1.7	1.825	2	5.525	7.525	4	172	420	226	31	17	0.425	0.137168	10.5
18	4	40	2	1.975	2	2.175	1.35	6.15	7.5	25	170	60	255	6	5	0.125	0.023529	1.5
8	4	40	2	1.925	2.05	1.775	2	5.75	7.75	6	189	109	196	12	8	0.2	0.061224	2.725
18	3	40	2	2.15	2.275	1.625	1.575	6.05	7.625	19	151	11	151	17	11	0.275	0.112583	0.275
8	2	40	2	2	1.975	1.375	2	5.35	7.35	8	166	21	383	9	6	0.15	0.023499	0.525
5	4	40	2	2.025	1.875	2.15	2	6.05	8.05	6	170	25	266	25	17	0.425	0.093985	0.625
14	2	40	2	1.975	1.95	1.6	2	5.525	7.525	10	171	97	368	37	13	0.325	0.100543	2.425
7	2	40	2	2	2	1.575	2	5.575	7.575	12	210	194	391	36	12	0.3	0.092072	4.85
3	3	40	2	1.85	2	2.1	2	5.95	7.95	52	171	566	251	45	27	0.675	0.179283	14.15
10	4	40	2	1.7	1.75	2	2	5.45	7.45	5	177	72	263	17	12	0.3	0.064639	1.8
6	3	40	2	2.075	1.7	1.75	2	5.525	7.525	33	192	183	271	28	16	0.4	0.103321	4.575
1	3	40	2	1.975	1.9	1.9	2	5.775	7.775	25	169	157	281	42	23	0.575	0.149466	3.925
9	3	40	2	1.9	1.575	1.7	2	5.175	7.175	5	196	138	249	27	13	0.325	0.108434	3.45
8	3	40	2	2.075	1.95	1.75	2.025	5.775	7.8	5	177	54	298	16	10	0.25	0.053691	1.35
6	4	40	2	2.1	2.175	1.8	2	6.075	8.075	10	168	53	219	19	14	0.35	0.086758	1.325
3	2	40	2	1.9	1.925	1.925	1.975	5.75	7.725	16	171	66	369	33	18	0.45	0.089431	1.65
5	3	40	2	2.075	1.95	1.875	2	5.9	7.9	33	167	138	340	31	18	0.45	0.091176	3.45
9	4	40	2	1.975	2.05	1.775	2	5.8	7.8	12	188	43	249	16	11	0.275	0.064257	1.075
10	3	40	2	1.775	1.85	1.875	2	5.5	7.5	40	163	190	284	25	15	0.375	0.088028	4.75
12	4	40	3	1.95	2.05	1.875	2	5.875	7.875	2	159	48	209	15	13	0.325	0.07177	1.2
20	3	40	3	1.8	2	1.85	1.375	5.65	7.025	36	158	66	206	20	14	0.35	0.097087	1.65
13	3	40	3	1.8	2.025	2.125	2	5.95	7.95	45	191	23	269	27	16	0.4	0.100372	0.575
15	4	40	3	2.025	2.025	2.075	2	6.125	8.125	4	178	322	355	24	16	0.4	0.067606	8.05
15	2	40	3	1.925	2	1.125	2	5.05	7.05	4	168	7	453	6	5	0.125	0.013245	0.175
2	2	40	3	1.975	2.125	1.5	2	5.6	7.6	19	165	282	308	29	14	0.35	0.094156	7.05
4	2	40	3	1.875	1.95	1.3	1.875	5.125	7	2	200	17	370	9	6	0.15	0.024324	0.425
19	2	40	3	2	1.85	1.75	1.05	5.6	6.65	22	186	316	425	27	12	0.3	0.063529	7.9
11	2	40	3	1.975	1.975	1.7	1.875	5.65	7.525	16	185	66	373	16	9	0.225	0.042895	1.65
4	4	40	3	2.025	2	2	2	6.025	8.025	14	213	24	259	6	5	0.125	0.023166	0.6
12	2	40	3	1.975	2.05	1.45	2	5.475	7.475	25	172	107	384	31	15	0.375	0.080729	2.675
13	4	40	3	1.975	1.975	2.125	2	6.075	8.075	3	188	45	230	20	13	0.325	0.086957	1.125
11	4	40	3	1.95	1.85	2.25	1.45	6.05	7.5	23	195	186	334	25	15	0.375	0.07485	4.65
16	2	40	3	2.125	2	2	2	6.125	8.125	11	184	130	346	12	7	0.175	0.034682	3.25
20	4	40	3	1.825	2.1	1.825	2	5.75	7.75	1	146	265	199	23	13	0.325	0.115578	6.625
20	2	40	3	1.9	2	1.575	1.075	5.475	6.55	13	180	81	358	21	12	0.3	0.058659	2.025
19	4	40	3	2.075	2	1.975	2	6.05	8.05	9	202	9	243	6	6	0.15	0.024691	0.225
17	2	40	3	1.923077	1.897436	1.051282	1	4.871795	5.871795	7	155	119	324	29	14	0.35	0.089506	2.975
4	3	40	3	2.225	1.55	1.8	1.175	5.575	6.75	28	171	112	217	27	14	0.35	0.124424	2.8
12	3	40	3	2.075	2.025	1.925	2	6.025	8.025	47	161	165	188	50	29	0.725	0.265957	4.125
17	3	40	3	1.825	2	2.025	1.5	5.85	7.35	25	179	79	288	42	21	0.525	0.145833	1.975
17	4	40	3	2.025	2.25	1.95	2	6.225	8.225	3	148	246	209	29	24	0.6	0.138756	6.15
16	4	40	3	2.1	2.025	2.15	2	6.275	8.275	16	177	66	263	18	13	0.325	0.068441	1.65
13	2	40	3	2.025641	1.948718	1.794872	1.923077	5.769231	7.692307	19	184	86	289	37	14	0.35	0.128028	2.15
2	4	40	3	1.95	2.025	1.8	2	5.775	7.775	1	183	16	223	12	12	0.3	0.053812	0.4
2	3	40	3	1.875	1.55	1.6	2	5.025	7.025	12	182	181	241	24	15	0.375	0.099585	4.525
15	3	40	3	2.1	2.275	1.575	2	5.95	7.95	32	187	4	186	14	12	0.3	0.075269	0.1
19	3	40	3	2.025	2.125	1.725	1.575	5.875	7.45	30	160	102	225	32	17	0.425	0.142222	2.55
11	3	40	3	2.025	2.175	1.775	1.925	5.975	7.9	28	178	51	176	25	18	0.45	0.142045	1.275
16	3	40	3	2.175	2.05	1.875	2	6.1	8.1	63	168	238	155	34	18	0.45	0.219355	5.95

Supplemental Table S1: CONSORT 2010 checklist of information to include when reporting a randomised trial*

Section/Topic	Item No	Checklist item	Reported on page No
**Title and abstract**			
	1a	Identification as a randomised trial in the title	Title
	1b	Structured summary of trial design, methods, results, and conclusions (for specific guidance see CONSORT for abstracts)	Abstract, paragraph 1-3
**Introduction**			
Background and objectives	2a	Scientific background and explanation of rationale	Introduction, paragraph 1-6
	2b	Specific objectives or hypotheses	Introduction, paragraph 7-8
**Methods**			
Trial design	3a	Description of trial design (such as parallel, factorial) including allocation ratio	Methods, paragraph 1
	3b	Important changes to methods after trial commencement (such as eligibility criteria), with reasons	none
Participants	4a	Eligibility criteria for participants	Methods, paragraph 2
	4b	Settings and locations where the data were collected	Methods, paragraph 1
Interventions	5	The interventions for each group with sufficient details to allow replication, including how and when they were actually administered	Methods, paragraph 2-5
Outcomes	6a	Completely defined pre-specified primary and secondary outcome measures, including how and when they were assessed	Methods, paragraph 7-8
	6b	Any changes to trial outcomes after the trial commenced, with reasons	none
Sample size	7a	How sample size was determined	Methods, paragraph 9-10
	7b	When applicable, explanation of any interim analyses and stopping guidelines	Methods,

			paragraph 21
Randomisation:			
Sequence generation	8a	Method used to generate the random allocation sequence	Methods, paragraph 11
	8b	Type of randomisation; details of any restriction (such as blocking and block size)	Methods, paragraph 11
Allocation concealment mechanism	9	Mechanism used to implement the random allocation sequence (such as sequentially numbered containers), describing any steps taken to conceal the sequence until interventions were assigned	Methods, paragraph 11
Implementation	10	Who generated the random allocation sequence, who enrolled participants, and who assigned participants to interventions	Methods, paragraph 11
Blinding	11a	If done, who was blinded after assignment to interventions (for example, participants, care providers, those assessing outcomes) and how	none
	11b	If relevant, description of the similarity of interventions	Not relevant
Statistical methods	12a	Statistical methods used to compare groups for primary and secondary outcomes	Methods, paragraph 18-20
	12b	Methods for additional analyses, such as subgroup analyses and adjusted analyses	Methods, paragraph 20
**Results**			
Participant flow (a diagram is strongly recommended)	13a	For each group, the numbers of participants who were randomly assigned, received intended treatment, and were analysed for the primary outcome	File S1
	13b	For each group, losses and exclusions after randomisation, together with reasons	File S1
Recruitment	14a	Dates defining the periods of recruitment and follow-up	File S1
	14b	Why the trial ended or was stopped	Results, paragraph 9
Baseline data	15	A table showing baseline demographic and clinical characteristics for each group	Table 2
Numbers analysed	16	For each group, number of participants (denominator) included in each analysis and whether the analysis was by original assigned groups	File S1
Outcomes and estimation	17a	For each primary and secondary outcome, results for each group, and the estimated effect size and its precision (such as 95% confidence interval)	Results, paragraph 2-8
	17b	For binary outcomes, presentation of both absolute and relative effect sizes is recommended	Results, paragraph 2-8

Ancillary analyses	18	Results of any other analyses performed, including subgroup analyses and adjusted analyses, distinguishing pre-specified from exploratory	none
Harms	19	All important harms or unintended effects in each group (for specific guidance see CONSORT for harms)	Results, paragraph 9
**Discussion**			
Limitations	20	Trial limitations, addressing sources of potential bias, imprecision, and, if relevant, multiplicity of analyses	Discussion, paragraph 8-9
Generalisability	21	Generalisability (external validity, applicability) of the trial findings	Discussion, paragraph 9
Interpretation	22	Interpretation consistent with results, balancing benefits and harms, and considering other relevant evidence	Discussion, paragraph 5-7
**Other information**			
Registration	23	Registration number and name of trial registry	Abstract, paragraph 4
Protocol	24	Where the full trial protocol can be accessed, if available	Methods, paragraph 2
Funding	25	Sources of funding and other support (such as supply of drugs), role of funders	Acknowledgm ents, paragraph 2

*We strongly recommend reading this statement in conjunction with the CONSORT 2010 Explanation and Elaboration for important clarifications on all the items. If relevant, we also recommend reading CONSORT extensions for cluster randomised trials, non-inferiority and equivalence trials, non-pharmacological treatments, herbal interventions, and pragmatic trials. Additional extensions are forthcoming: for those and for up to date references relevant to this checklist, see www.consort-statement.org.

Supplemental Table S2: Remaining Secondary Outcome Tables

	**House Index**		
	Control	Guppies	Guppies + PPF
Baseline (range)	40% (18-58)	54% (33-73)	42% (23-78)
Dry Season (range)	36% (10-45)	23% (13-45)	27% (15-38)
Light Rain (range)	71% (45-90)	43% (25-68)	44% (30-73)
Heavy Rain (range)	37% (13-65)	33% (13-60)	33% (13-60)
Total (range)	48% (10-90)	33% (13-68)	34% (13-73)
Ratio (95% CI), p-value*	1 (ref)	0.54 (0.45-0.63), p<0.001	0.58 (0.49-0.68), p<0.001
Ratio (95% CI), p-value*	**	1 (ref)	1.07 (0.91-1.27), p=0.411
	**Container Index**		
	Control	Guppies	Guppies + PPF
Baseline (range)	16% (5-38)	20% (8-31)	17% (6-35)
Dry Season (range)	10% (3-14)	5% (1-10)	6% (2-13)
Light Rain (range)	21% (10-39)	12% (5-21)	14% (8-27)
Heavy Rain (range)	10% (3-19)	9% (2-17)	7% (2-14)
Total (range)	13% (3-39)	8% (1-21)	8% (2-27)
Ratio (95% CI), p-value*	1 (ref)	0.61 (0.55-0.67), p<0.001	0.61 (0.55-0.67), p<0.001
Ratio (95% CI), p-value*	**	1 (ref)	1.00 (0.90-1.11), p=0.991
	**Pupae Per House**		
	Control	Guppies	Guppies + PPF
Baseline (range)	4.2 (1.0-11.6)	17.8 (0.8-69.4)	5.3 (2.4-11.4)
Dry Season (range)	4.8 (0.5-16.7)	1.6 (0.2-4.9)	3.0 (0.2-7.9)
Light Rain (range)	9.8 (2.5-32.9)	5.4 (0.3-14.2)	2.6 (0.1-6.0)
Heavy Rain (range)	3.1 (0.4-8.9)	2.7 (0.6-11.7)	3.1 (0.4-8.1)

Total (range)	5.9 (0.4-32.9)	3.2 (0.2-14.2)	2.9 (0.1-8.1)
Ratio (95% CI), p-value*	1 (ref)	0.55 (0.34-0.88), p=0.0130	0.49 (0.30-0.79), p=0.0032
Ratio (95% CI), p-value*	**	1 (ref)	0.89 (0.55-1.44), p=0.642
	**Know Dengue is Transmitted by *Aedes* Mosquitoes**		
	Control	Guppies	PPF + Guppies
Baseline (%, 95% CI)	98% (96.0-1.00)	95.5% (92.6-98.4)	97.0% (94.6-99.4)
Endline (%, 95% CI)	100% (NA)	100% (NA)	100% (NA)
Ratio (95% CI), p-value	1 (ref)	0.99 (0.86-1.14), p=0.915	1.01 (0.87-1.16), p=0.943

*The ratios do not include the baseline data

**The ratios are not given here as they are redundant
