# Supplementary material for: Field Efficacy of Larvivorous Fish and Pyriproxyfen Combined with Community Engagement on Dengue Vectors in Cambodia: A Randomized Controlled Trial
Source: Am J Trop Med Hyg. 2021 Sep 7;105(5):1265–76. doi: 10.4269/ajtmh.20-1088 (PMC8592206; doi:10.4269/ajtmh.20-1088)
Supplement: Supplementary file 3 [file tpmd201088.SD3.pdf]

cluster	survey	house	arm	_2house	_2yard	_2shade	_2water	pci3	pci4	aedes_fem	hhmember	pupae_wei	cont_total	cont_positi	house_pos	houseinde	containerir	pupaeperhouse
29	4	40	1	1.925	1.85	1.975	2	5.75	7.75	7	177	116	319	22	14	0.35	0.068966	2.9
30	2	40	1	2	2	1.3	2	5.3	7.3	8	165	257	357	30	14	0.35	0.084034	6.425
29	2	40	1	1.9	2.025	1.375	2	5.3	7.3	31	200	666	427	61	16	0.4	0.142857	16.65
24	3	40	1	1.9	1.975	1.775	2	5.65	7.65	56	189	1317	302	64	28	0.7	0.211921	32.925
26	2	40	1	1.975	1.925	1.725	2	5.625	7.625	14	210	298	417	46	18	0.45	0.110312	7.45
27	3	40	1	2.225	2	2.025	2	6.25	8.25	57	200	501	264	45	29	0.725	0.170455	12.525
21	4	40	1	1.975	1.925	1.8	2	5.7	7.7	11	154	39	229	26	16	0.4	0.113537	0.975
24	4	40	1	2	2.025	2.075	2.025	6.1	8.125	23	210	17	233	12	9	0.225	0.051502	0.425
29	3	40	1	1.9	1.975	2.125	2	6	8	134	192	100	260	57	30	0.75	0.219231	2.5
23	2	40	1	2.1	2.025	1.575	2	5.7	7.7	15	179	20	263	7	4	0.1	0.026616	0.5
30	3	40	1	2.15	2.125	1.925	2.025	6.2	8.225	64	163	450	191	75	38	0.95	0.39267	11.25
23	4	40	1	2.05	2.175	1.925	2	6.15	8.15	7	174	139	191	24	19	0.475	0.125654	3.475
25	3	40	1	2.05	1.95	2.05	2	6.05	8.05	181	181	380	275	44	27	0.675	0.16	9.5
23	3	40	1	2	1.575	1.6	2	5.175	7.175	17	188	156	204	36	23	0.575	0.176471	3.9
25	2	40	1	1.975	2	1.375	2	5.35	7.35	19	165	115	368	15	11	0.275	0.040761	2.875
30	4	40	1	1.9	1.875	2.2	2	5.975	7.975	15	169	355	323	60	26	0.65	0.185759	8.875
26	4	40	1	2.05	2	2	2	6.05	8.05	6	192	176	236	13	10	0.25	0.055085	4.4
27	2	40	1	2.3	2.05	1.675	2	6.025	8.025	36	189	181	412	45	20	0.5	0.109223	4.525
27	4	40	1	1.975	2	2	2	5.975	7.975	10	200	161	251	8	5	0.125	0.031873	4.025
26	3	40	1	2.15	2.1	1.85	2	6.1	8.1	74	164	352	180	51	29	0.725	0.283333	8.8
22	4	40	1	1.975	2.15	1.675	2	5.8	7.8	8	187	91	220	21	14	0.35	0.095455	2.275
24	2	40	1	2.051282	2.128205	1.384615	2	5.564103	7.564103	49	198	52	348	35	17	0.425	0.100575	1.3
21	3	40	1	2.025	1.9	1.825	2	5.75	7.75	37	155	100	291	29	18	0.45	0.099656	2.5
22	2	40	1	2.05	2.075	1.425	2	5.55	7.55	3	194	103	333	22	10	0.25	0.066066	2.575
22	3	40	1	2.125	1.65	1.675	2	5.45	7.45	46	211	306	212	45	24	0.6	0.212264	7.65
25	4	40	1	2	2.475	1.625	2	6.1	8.1	5	163	106	194	23	17	0.425	0.118557	2.65
28	3	40	1	2.25	1.975	1.95	2	6.175	8.175	86	160	260	249	74	36	0.9	0.297189	6.5
28	4	40	1	2.125	2.2	1.75	2.025	6.075	8.1	4	139	45	233	24	17	0.425	0.103004	1.125
28	2	40	1	1.974359	2.025641	1.815789	2	5.81579	7.81579	16	151	174	414	60	20	0.5	0.144928	4.35
21	2	40	1	2	2.025	1.225	2	5.25	7.25	6	137	55	281	29	12	0.3	0.103203	1.375
10	2	40	2	1.95	1.875	1.975	2	5.8	7.8	17	159	50	344	18	10	0.25	0.052326	1.25
14	4	40	2	1.975	2.2	1.725	2	5.9	7.9	2	158	88	227	23	15	0.375	0.101322	2.2
18	2	40	2	2.2	2.075	1.25	2	5.525	7.525	6	184	22	310	8	6	0.15	0.025806	0.55
7	4	40	2	1.975	1.75	2.225	2	5.95	7.95	23	195	104	252	42	24	0.6	0.166667	2.6
5	2	40	2	2.025	2	1.825	2	5.85	7.85	11	178	1	384	5	3	0.075	0.013021	0.025
14	3	40	2	1.925	2	1.975	2	5.9	7.9	71	189	311	282	60	20	0.5	0.212766	7.775
6	2	40	2	2	2	1.275	2	5.275	7.275	3	193	66	337	16	8	0.2	0.047478	1.65
1	4	40	2	1.95	1.725	2.075	2	5.75	7.75	8	189	39	230	14	8	0.2	0.06087	0.975
3	4	40	2	1.875	2.075	1.875	1.875	5.825	7.7	19	163	469	192	29	19	0.475	0.151042	11.725
9	2	40	2	1.975	1.975	1.1	2	5.05	7.05	10	192	6	373	7	5	0.125	0.018767	0.15
1	2	40	2	2.05	2	1.5	2	5.55	7.55	14	180	121	360	21	9	0.225	0.058333	3.025
7	3	40	2	2	1.7	1.825	2	5.525	7.525	4	172	420	226	31	17	0.425	0.137168	10.5
18	4	40	2	1.975	2	2.175	1.35	6.15	7.5	25	170	60	255	6	5	0.125	0.023529	1.5
8	4	40	2	1.925	2.05	1.775	2	5.75	7.75	6	189	109	196	12	8	0.2	0.061224	2.725
18	3	40	2	2.15	2.275	1.625	1.575	6.05	7.625	19	151	11	151	17	11	0.275	0.112583	0.275
8	2	40	2	2	1.975	1.375	2	5.35	7.35	8	166	21	383	9	6	0.15	0.023499	0.525
5	4	40	2	2.025	1.875	2.15	2	6.05	8.05	6	170	25	266	25	17	0.425	0.093985	0.625
14	2	40	2	1.975	1.95	1.6	2	5.525	7.525	10	171	97	368	37	13	0.325	0.100543	2.425
7	2	40	2	2	2	1.575	2	5.575	7.575	12	210	194	391	36	12	0.3	0.092072	4.85
3	3	40	2	1.85	2	2.1	2	5.95	7.95	52	171	566	251	45	27	0.675	0.179283	14.15
10	4	40	2	1.7	1.75	2	2	5.45	7.45	5	177	72	263	17	12	0.3	0.064639	1.8
6	3	40	2	2.075	1.7	1.75	2	5.525	7.525	33	192	183	271	28	16	0.4	0.103321	4.575
1	3	40	2	1.975	1.9	1.9	2	5.775	7.775	25	169	157	281	42	23	0.575	0.149466	3.925
9	3	40	2	1.9	1.575	1.7	2	5.175	7.175	5	196	138	249	27	13	0.325	0.108434	3.45
8	3	40	2	2.075	1.95	1.75	2.025	5.775	7.8	5	177	54	298	16	10	0.25	0.053691	1.35
6	4	40	2	2.1	2.175	1.8	2	6.075	8.075	10	168	53	219	19	14	0.35	0.086758	1.325
3	2	40	2	1.9	1.925	1.925	1.975	5.75	7.725	16	171	66	369	33	18	0.45	0.089431	1.65
5	3	40	2	2.075	1.95	1.875	2	5.9	7.9	33	167	138	340	31	18	0.45	0.091176	3.45
9	4	40	2	1.975	2.05	1.775	2	5.8	7.8	12	188	43	249	16	11	0.275	0.064257	1.075
10	3	40	2	1.775	1.85	1.875	2	5.5	7.5	40	163	190	284	25	15	0.375	0.088028	4.75
12	4	40	3	1.95	2.05	1.875	2	5.875	7.875	2	159	48	209	15	13	0.325	0.07177	1.2
20	3	40	3	1.8	2	1.85	1.375	5.65	7.025	36	158	66	206	20	14	0.35	0.097087	1.65
13	3	40	3	1.8	2.025	2.125	2	5.95	7.95	45	191	23	269	27	16	0.4	0.100372	0.575
15	4	40	3	2.025	2.025	2.075	2	6.125	8.125	4	178	322	355	24	16	0.4	0.067606	8.05
15	2	40	3	1.925	2	1.125	2	5.05	7.05	4	168	7	453	6	5	0.125	0.013245	0.175
2	2	40	3	1.975	2.125	1.5	2	5.6	7.6	19	165	282	308	29	14	0.35	0.094156	7.05
4	2	40	3	1.875	1.95	1.3	1.875	5.125	7	2	200	17	370	9	6	0.15	0.024324	0.425
19	2	40	3	2	1.85	1.75	1.05	5.6	6.65	22	186	316	425	27	12	0.3	0.063529	7.9
11	2	40	3	1.975	1.975	1.7	1.875	5.65	7.525	16	185	66	373	16	9	0.225	0.042895	1.65
4	4	40	3	2.025	2	2	2	6.025	8.025	14	213	24	259	6	5	0.125	0.023166	0.6
12	2	40	3	1.975	2.05	1.45	2	5.475	7.475	25	172	107	384	31	15	0.375	0.080729	2.675
13	4	40	3	1.975	1.975	2.125	2	6.075	8.075	3	188	45	230	20	13	0.325	0.086957	1.125
11	4	40	3	1.95	1.85	2.25	1.45	6.05	7.5	23	195	186	334	25	15	0.375	0.07485	4.65
16	2	40	3	2.125	2	2	2	6.125	8.125	11	184	130	346	12	7	0.175	0.034682	3.25
20	4	40	3	1.825	2.1	1.825	2	5.75	7.75	1	146	265	199	23	13	0.325	0.115578	6.625
20	2	40	3	1.9	2	1.575	1.075	5.475	6.55	13	180	81	358	21	12	0.3	0.058659	2.025
19	4	40	3	2.075	2	1.975	2	6.05	8.05	9	202	9	243	6	6	0.15	0.024691	0.225
17	2	40	3	1.923077	1.897436	1.051282	1	4.871795	5.871795	7	155	119	324	29	14	0.35	0.089506	2.975
4	3	40	3	2.225	1.55	1.8	1.175	5.575	6.75	28	171	112	217	27	14	0.35	0.124424	2.8
12	3	40	3	2.075	2.025	1.925	2	6.025	8.025	47	161	165	188	50	29	0.725	0.265957	4.125
17	3	40	3	1.825	2	2.025	1.5	5.85	7.35	25	179	79	288	42	21	0.525	0.145833	1.975
17	4	40	3	2.025	2.25	1.95	2	6.225	8.225	3	148	246	209	29	24	0.6	0.138756	6.15
16	4	40	3	2.1	2.025	2.15	2	6.275	8.275	16	177	66	263	18	13	0.325	0.068441	1.65
13	2	40	3	2.025641	1.948718	1.794872	1.923077	5.769231	7.692307	19	184	86	289	37	14	0.35	0.128028	2.15
2	4	40	3	1.95	2.025	1.8	2	5.775	7.775	1	183	16	223	12	12	0.3	0.053812	0.4
2	3	40	3	1.875	1.55	1.6	2	5.025	7.025	12	182	181	241	24	15	0.375	0.099585	4.525
15	3	40	3	2.1	2.275	1.575	2	5.95	7.95	32	187	4	186	14	12	0.3	0.075269	0.1
19	3	40	3	2.025	2.125	1.725	1.575	5.875	7.45	30	160	102	225	32	17	0.425	0.142222	2.55
11	3	40	3	2.025	2.175	1.775	1.925	5.975	7.9	28	178	51	176	25	18	0.45	0.142045	1.275
16	3	40	3	2.175	2.05	1.875	2	6.1	8.1	63	168	238	155	34	18	0.45	0.219355	5.95
